# Dynamics of Chloroplast Translation during Chloroplast Differentiation in Maize

**DOI:** 10.1371/journal.pgen.1006106

**Published:** 2016-07-14

**Authors:** Prakitchai Chotewutmontri, Alice Barkan

**Affiliations:** Institute of Molecular Biology, University of Oregon, Eugene, Oregon, United States of America; Institut de Biologie Physico-Chimique, FRANCE

## Abstract

Chloroplast genomes in land plants contain approximately 100 genes, the majority of which reside in polycistronic transcription units derived from cyanobacterial operons. The expression of chloroplast genes is integrated into developmental programs underlying the differentiation of photosynthetic cells from non-photosynthetic progenitors. In C4 plants, the partitioning of photosynthesis between two cell types, bundle sheath and mesophyll, adds an additional layer of complexity. We used ribosome profiling and RNA-seq to generate a comprehensive description of chloroplast gene expression at four stages of chloroplast differentiation, as displayed along the maize seedling leaf blade. The rate of protein output of most genes increases early in development and declines once the photosynthetic apparatus is mature. The developmental dynamics of protein output fall into several patterns. Programmed changes in mRNA abundance make a strong contribution to the developmental shifts in protein output, but output is further adjusted by changes in translational efficiency. RNAs with prioritized translation early in development are largely involved in chloroplast gene expression, whereas those with prioritized translation in photosynthetic tissues are generally involved in photosynthesis. Differential gene expression in bundle sheath and mesophyll chloroplasts results primarily from differences in mRNA abundance, but differences in translational efficiency amplify mRNA-level effects in some instances. In most cases, rates of protein output approximate steady-state protein stoichiometries, implying a limited role for proteolysis in eliminating unassembled or damaged proteins under non-stress conditions. Tuned protein output results from gene-specific trade-offs between translational efficiency and mRNA abundance, both of which span a large dynamic range. Analysis of ribosome footprints at sites of RNA editing showed that the chloroplast translation machinery does not generally discriminate between edited and unedited RNAs. However, editing of ACG to AUG at the *rpl2* start codon is essential for translation initiation, demonstrating that ACG does not serve as a start codon in maize chloroplasts.

## Author Summary

Chloroplasts are subcellular organelles in plants and algae that carry out the core reactions of photosynthesis. Chloroplasts originated as cyanobacterial endosymbionts. Subsequent coevolution with their eukaryotic host resulted in a massive transfer of genes to the nuclear genome, the acquisition of new gene expression mechanisms, and the integration of chloroplast functions into host programs. Chloroplasts in multicellular plants develop from non-photosynthetic proplastids, a process that involves a prodigious increase in the expression of chloroplast genes encoding components of the photosynthetic apparatus. We used RNA sequencing and ribosome profiling to generate a comprehensive description of the dynamics of chloroplast gene expression during the transformation of proplastids into the distinct chloroplast types found in bundle sheath and mesophyll cells in maize. Genes encoding proteins that make up the chloroplast gene expression machinery peak in protein output earlier in development than do those encoding proteins that function in photosynthesis. Programmed changes in translational efficiencies superimpose on changes in mRNA abundance to shift the balance of protein output as chloroplast development proceeds. We also mined the data to gain insight into general features of chloroplast gene expression, such as relative translational efficiencies, the impact of RNA editing on translation, and the identification of rate limiting steps in gene expression. The findings clarify the parameters that dictate the abundance of chloroplast gene products and revealed unanticipated phenomena to be addressed in future studies.

## Introduction

The evolution of chloroplasts from a cyanobacterial endosymbiont was accompanied by a massive transfer of bacterial genes to the nuclear genome, and by the integration of chloroplast processes into the host’s developmental and physiological programs [1]. In multicellular plants, chloroplasts differentiate from non-photosynthetic proplastids in concert with the differentiation of meristematic cells into photosynthetic leaf cells. This transformation is accompanied by a prodigious increase in the abundance of the proteins that make up the photosynthetic apparatus, which contribute more than half of the protein mass in photosynthetic leaf tissue [2]. Both nuclear and chloroplast genes contribute subunits to the multisubunit complexes that participate in photosynthesis. The expression of these two physically separated gene sets is coordinated by nucleus-encoded proteins that control chloroplast gene expression, and by signals emanating from chloroplasts that influence nuclear gene expression [1, 3]. Beyond these general concepts, however, little is known about the mechanisms that coordinate chloroplast and nuclear gene expression in the context of the proplastid to chloroplast transition. Furthermore, a thorough description of the dynamics of chloroplast gene expression during this process is currently lacking.

Despite roughly one billion years of evolution, the bacterial ancestry of the chloroplast genome is readily apparent in its gene organization and gene expression mechanisms. Most chloroplast genes in land plants are grouped into polycistronic transcription units [4] that are transcribed by a bacterial-type RNA polymerase [5] and translated by 70S ribosomes that strongly resemble bacterial ribosomes [6]. As in bacteria, chloroplast ribosomes bind mRNA at ribosome binding sites near start codons, sometimes with the assistance of a Shine-Dalgarno element [6]. Superimposed on this ancient scaffold are numerous features that arose post-endosymbiosis [7]. For example, a phage-type RNA polymerase collaborates with an RNA polymerase of cyanobacterial origin [5], and chloroplast RNAs are modified by RNA editing, RNA splicing, and other events that are either unusual or absent in bacteria [8].

Ribosome profiling data from *E*. *coli* revealed that the rate of protein output from genes encoding subunits of multisubunit complexes is proportional to subunit stoichiometry, and that proportional synthesis is typically achieved by differences in the translational efficiency of genes residing in the same operon [9, 10]. As the majority of chloroplast gene products are components of multisubunit complexes, it is of interest to know whether similar themes apply. Furthermore, the gene content of polycistronic transcription units in chloroplasts has diverged from that in the cyanobacterial ancestor. Has “tuned” protein output been maintained in chloroplasts despite this disrupted operon organization? If so, what mechanisms achieve this tuning in light of the new gene arrangements and the new features of mRNA metabolism?

In this work, we used ribosome profiling to address these and other questions of chloroplast gene regulation in the context of the proplastid to chloroplast transition. For this purpose, we took advantage of the natural developmental gradient of the maize seedling leaf blade, where cells and plastids at increasing stages of photosynthetic differentiation form a developmental gradient from base to tip [11]. By using the normalized abundance of ribosome footprints as a proxy for rates of protein synthesis, we show that the rate of protein output from many chloroplast genes is tuned to protein stoichiometry, and that tuned protein output is achieved through gene-specific balancing of mRNA abundance with translational efficiency. This comprehensive analysis revealed developmentally programmed changes in translational efficiencies, which superimpose on programmed changes in mRNA abundance to shift the balance of protein output as chloroplast development proceeds.

## Results

### Experimental design

**Fig 1:**
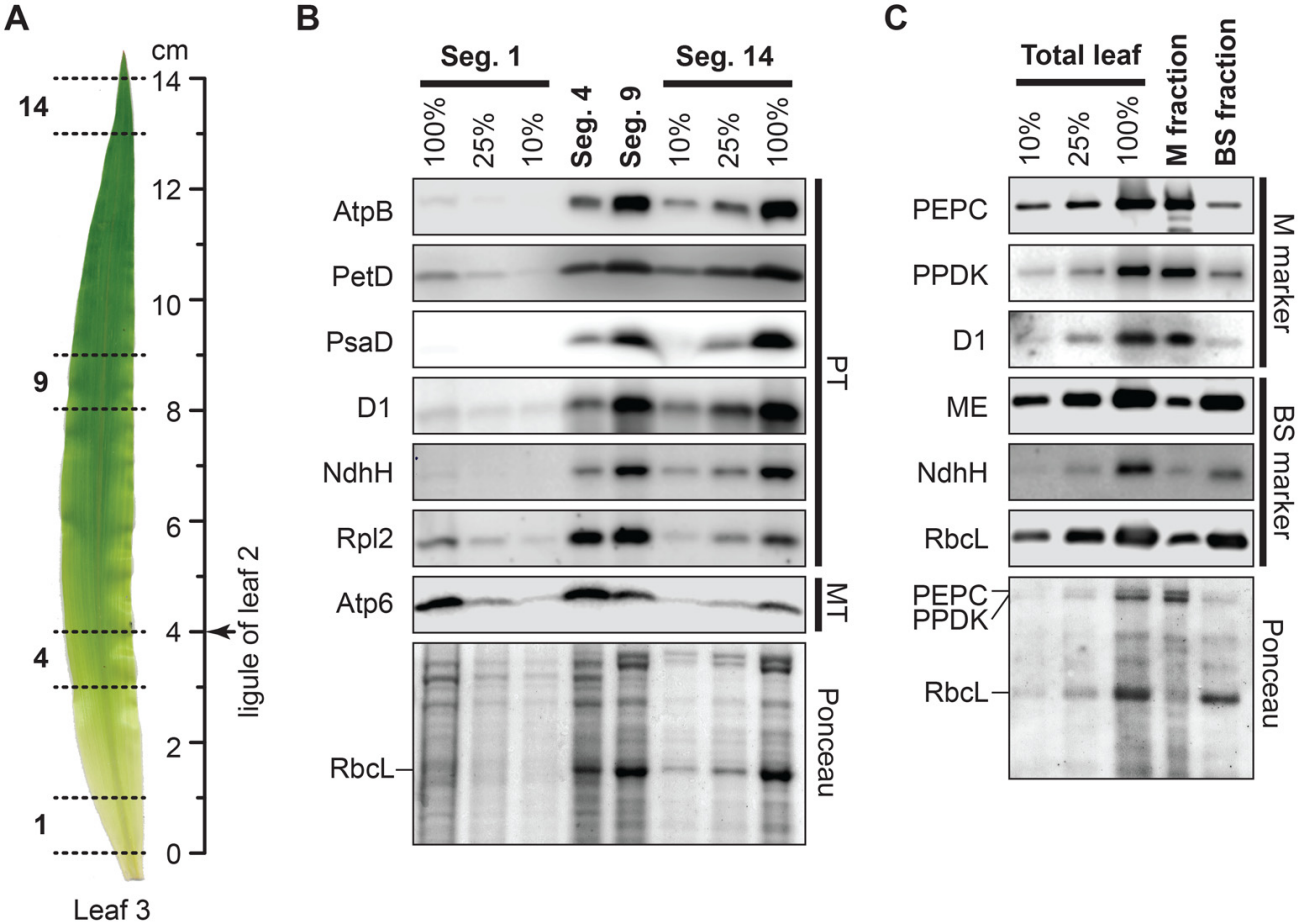
Tissue samples used for ribosome profiling. **(A)** Leaf segments analyzed in this study. The indicated segments were excised from leaf 3 of nine-day-old seedlings. The segments are numbered according to the nomenclature of [13]. **(B)** Immunoblots showing abundance of marker proteins in the leaf gradient tissue used for ribosome profiling. Replicate immunoblots were probed with antibodies to subunits of the chloroplast ATP synthase (AtpB), cytochrome *b*_*6*_*f* complex (PetD), Photosystem I (PsaD), Photosystem II (D1), the NDH-like complex NdhH), chloroplast ribosomes (Rpl2), and mitochondrial ATP synthase (Atp6). Samples were loaded on the basis of equal protein. An image of one blot stained with Ponceau S (below) serves as a loading control and illustrates the abundance of the large subunit of Rubisco (RbcL). PT, plastid proteins; MT, mitochondrial protein. **(C**) Replicate immunoblots showing abundance of marker proteins in mesophyll (M) and bundle sheath (BS) fractions used for ribosome profiling. Samples were loaded on the basis of equal protein. An image of one blot stained with Ponceau S (below) illustrates the abundance of several marker proteins. PEPC, phosphoenolpyruvate carboxylase; PPDK, pyruvate orthophosphate dikinase; ME, NADP-dependent malic enzyme; other abbreviations as in panel (B).

We analyzed tissues from the same genetic background and developmental stage as used in previous proteome [2] and nuclear transcriptome [12, 13] studies of photosynthetic differentiation in maize. Four leaf sections were harvested from the third leaf to emerge in 9-day old seedlings (Fig 1A): the leaf base (segment 1), which harbors non-photosynthetic proplastids; 3–4 cm above the base (segment 4), representing the sink-source transition and a region of active chloroplast biogenesis; 8–9 cm above the base (segment 9), representing young chloroplasts; and a section near the tip (segment 14) harboring mature bundle sheath and mesophyll chloroplasts [2, 12]. The developmental transitions represented by these fractions are illustrated in the immunoblot assays shown in Fig 1B. The mitochondrial protein Atp6 is most abundant in the two basal sections, subunits of photosynthetic complexes (AtpB, PetD, PsaD, PsbA, NdhH, RbcL) are most abundant in the two apical sections, and a chloroplast ribosomal protein (Rpl2) exhibits peak abundance in the two middle sections. These developmental profiles are consistent with prior proteome data [2].

To explore the contribution of differential chloroplast gene expression to the distinct proteomes in bundle sheath and mesophyll cells, we also analyzed bundle sheath and mesophyll-enriched fractions from the apical region of seedling leaves. Standard protocols for the separation of bundle sheath and mesophyll cells involve lengthy incubations that are likely to cause changes in ribosome position. We used a rapid mechanical fractionation method that minimizes the time between tissue disruption and the generation of ribosome footprints (see Materials and Methods). Markers for each cell type were enriched 5- to 10-fold in the corresponding fraction (Fig 1C). This degree of enrichment is comparable to that of the fractions used to define mesophyll and bundle sheath-enriched proteomes in maize [14].

We modified our previous method for preparing ribosome footprints from maize leaf tissue [15] to reduce the amount of time and tissue required, and to reduce contamination by non-ribosomal ribonucleoprotein particles (RNPs). In brief, leaf tissue was flash frozen and ground in liquid N_2_, thawed in a standard polysome extraction buffer, and treated with Ribonuclease I to liberate monosomes. Ribosomes were purified by pelleting through a sucrose cushion under conditions that leave chloroplast group II intron RNPs (~600 kDa) [16] in the supernatant (S1A Fig). RNAs between approximately 20 and 35 nucleotides (nt) were gel purified and converted to a sequencing library with a commercial small RNA library kit that has minimal ligation bias [17]. rRNA contaminants were depleted after first strand cDNA synthesis by hybridization to biotinylated oligonucleotides designed to match abundant contaminants detected in pilot experiments (S1 Table). Approximately 35 million reads were obtained for each “Ribo-seq” replicate, roughly 50% of which aligned to mRNA (S2 Table). RNA-seq data was generated from RNA extracted from aliquots of each lysate taken prior to addition of RNAse I. Replicate RNA-seq and Ribo-seq assays showed high reproducibility (Pearson correlation of >0.98, S2 Fig). Almost all plastid genes were represented by at least 100 reads per replicate in all datasets (S3 Fig). Several clusters of low abundance reads mapped to small unannotated ORFs, but further investigation is required to evaluate which, if any, of these are the footprints of translating ribosomes.

### Characteristics of ribosome footprints in the chloroplast, mitochondrion, and cytosol

**Fig 2:**
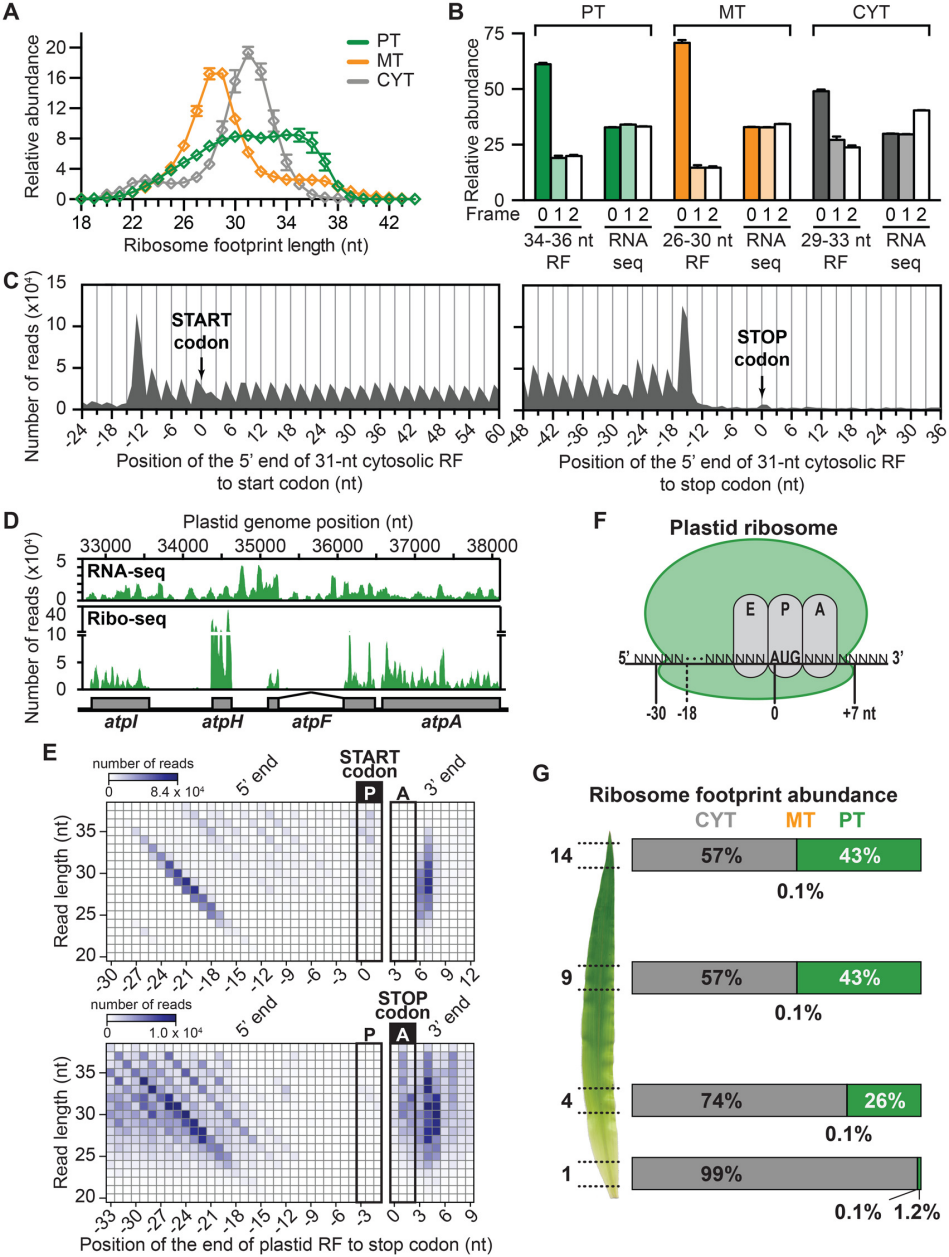
Characteristics of ribosome footprints from the cytosol (CYT), plastids (PT), and mitochondria (MT). **(A)** Size distribution of ribosome footprints. Values are the mean ± Standard Error of the Mean (SEM) from the twelve leaf segment datasets. **(B)** Three-nucleotide periodicity of Ribo-seq data. Histograms show the abundance of reads representing P-site placement in each of the three reading frames. P-site placements were inferred based on the footprint size and the size-dependent P-site positions inferred from our data (see panel **E** and S4 Fig). Values are the mean ± SEM from the 12 leaf segment datasets. **(C)** Meta-analysis of cytosolic ribosome footprints mapping near the start and stop codons of cytosolic ORFs in the leaf gradient datasets. Values show the number of 31-nt footprints with 5’ ends at each position. **(D)** Example of Ribo-seq and RNA-seq coverage in a chloroplast polycistronic transcription unit. Reads are combined from the twelve leaf segment datasets. The group II intron in the *atpF* gene is marked. **(E)** Meta-analysis of plastid ribosome footprints that map to start and stop codons. The data from the twelve leaf gradient datasets are parsed by footprint size. **(F)** Placement of plastid ribosome footprints with respect to the E, P, and A sites of the ribosome. The 5’-end placement varies with footprint size, while the 3’-end is constant at 7-nt downstream from the start of the P site (see data in panel **E**). **(G)** Partitioning of translational output among the three genetic compartments during photosynthetic differentiation. Values represent the average from three biological replicates. Note that the footprints from mitochondrial ribosomes may be under-represented due to the protocol used here.

Ribosomes in the cytosol, mitochondria, and chloroplasts have distinct genetic origins. Accordingly, the ribosome footprints from each compartment displayed different size distributions (Fig 2A). The cytosolic ribosome footprints showed a minor peak at 23 nucleotides and a major peak at 31 nucleotides, similar to observations in yeast [18]. The mitochondrial data showed a major peak at 28–29 nucleotides and a minor peak at 36 nucleotides, similar to the 27 and 33-nt peaks reported for human mitochondria [19]. The plastid ribosome footprints had a broad size distribution suggestive of two populations, with peaks at approximately 30 and 35 nucleotides. A similar distribution was observed in pilot experiments involving the gel purification of RNAs up to 40-nt (S1B Fig) indicating that the peak at 35-nt was not an artifact of our gel purification strategy. A broad and bimodal size distribution was also observed for chloroplast ribosome footprints from the single-celled alga *Chlamydomonas reinhardtii*, albeit with peaks at slightly different positions [20]. The two prior reports of ribosome footprint size distributions in plants [21, 22] did not parse the data from the three compartments, but the 31-nucleotide modal size reported in those studies is consistent with our data. Our data show the 3-nucleotide periodicity expected for ribosome footprints (Fig 2B and 2C). Interestingly, the degree of periodicity varies with footprint size (S4 Fig). The reads are largely restricted to open reading frames in the cytosol (Fig 2C) and chloroplast (Fig 2D). Taken together, these results provide strong evidence that the vast majority of the Ribo-seq reads come from bona-fide ribosome footprints.

The placement of ribosome P and A sites with respect to ribosome footprint termini has not been reported for any organellar ribosomes or for cytosolic ribosomes in maize. A meta analysis of our data showed that the position of the 3’ end of ribosome footprints from initiating and terminating ribosomes in chloroplasts and mitochondria is constant with respect to start and stop codons, respectively, regardless of footprint size; however, the position of the 5’ ends varies with footprint size (Fig 2E, S4C Fig). Therefore, the positions of the A and P sites in organellar ribosomes can be inferred based on the 3’-ends of their footprints, as is also true for bacterial ribosomes [23, 24]. The modal distance between the start of the P site in chloroplast ribosomes and the 3’-ends of chloroplast ribosome footprints is 7 nucleotides. By contrast, cytosolic ribosome footprints are approximately centered on the P site regardless of footprint size (S4B Fig).

The partitioning of ribosome footprints among the three genetic compartments shifts dramatically during the course of leaf development (Fig 2G). The contribution of cytosolic translation drops from 99% at the leaf base to 57% in the apical leaf sections due to the increasing contribution of ribosome footprints from chloroplasts. This shift of cellular resources towards chloroplast translation corresponds with the massive increase in the content of photosynthetic complexes harboring plastid-encoded subunits (Rubisco, PSII, PSI, cytochrome *b*_*6*_*f*, ATP synthase, NDH) (Fig 1). Ribosome footprints from mitochondria accounted for a very small fraction of the total at all stages. However, our protocol was not optimized for the quantitative recovery of mitochondrial ribosomes so these data may not reflect the total mitochondrial ribosome population.

### The translational output of most chloroplast genes is tuned to the stoichiometry of their products

In the discussion below we define the “translational output” of a gene as the abundance of ribosome footprints per kb per million reads mapped to nuclear coding sequences (RPKM), and we use this value to compare rates of protein synthesis among genes on a molar basis. This is a typical interpretation of Ribo-seq data, and it is based on evidence that the bulk rate of translation elongation on all ORFs is similar under any particular condition, despite the fact that ribosome pausing can lead to the over-representation of ribosomes at specific positions [9, 25]. Although this may be an over simplification in some instances, this interpretation of our data produced results that are generally coherent with current understanding of chloroplast biogenesis (see below). Group II introns interrupt eight protein-coding genes in maize chloroplasts. These present a challenge for data analysis because the unspliced transcripts make up a substantial fraction of the RNA pool [16] and translation can initiate on unspliced RNAs and terminate within introns [15]. We therefore calculated translational output based solely on the last exon (normalized to exon length). Data summaries presented below include RNA-seq data only for that subset of intron-containing genes for which multiple methods of analysis provided consistent values for the abundance of spliced RNA isoforms (see Materials and Methods).

**Fig 3:**
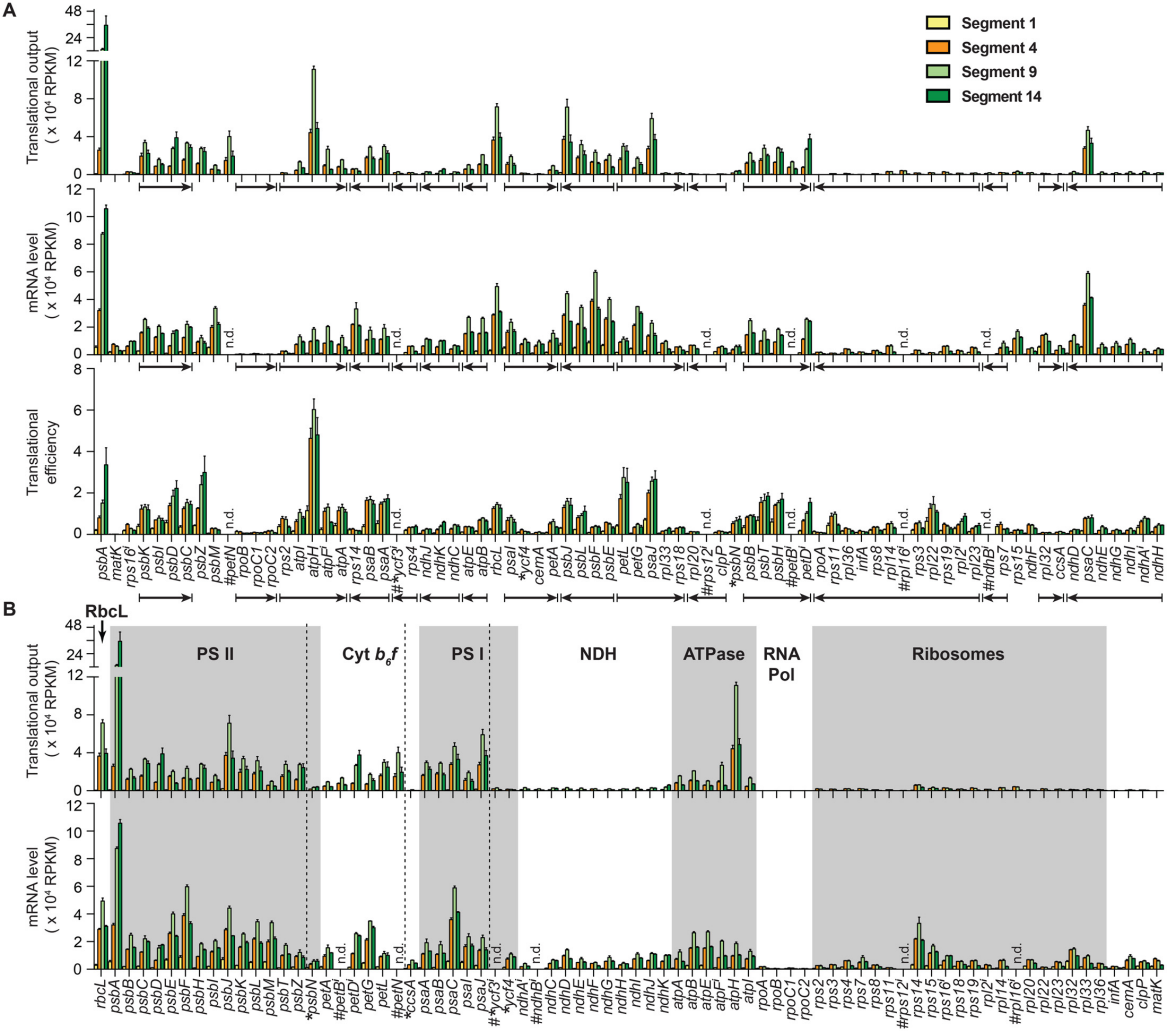
Genome-wide views of the plastid translatome and transcriptome during the proplastid to chloroplast transition. The data are expressed as reads per kilobase per million reads mapping to nuclear genome coding sequences (RPKM). Translational output is defined as Ribo-seq RPKM. mRNA level is defined as RNA-seq RPKM. Translational efficiency is calculated as the ratio of translational output to mRNA level. Values are the mean ± SEM from three replicates. Intron-containing genes are marked with a superscript *i*. Genes for which RNA levels and translational efficiency were not determined (n.d.) are marked (#). These include intron-containing genes for which the fraction of reads derived from spliced transcripts is uncertain, and *petN*, whose short mRNA is not represented quantitatively in the RNA-seq data. Genes encoding assembly factors are marked with asterisks. Other genes encode structural components of the complexes indicated in panel B. **(A)** Translational output, RNA abundance and translational efficiency displayed according to native gene order. Co-transcribed genes are marked with arrows that indicate the direction of transcription. **(B)** Translational output and RNA abundance displayed according to gene function. Genes encoding assembly factors are demarcated from the structural genes with dashed lines. The data for each functional group are plotted separately in Fig 4 and S6 Fig using Y-axis scales suited for the relevant values. The data for Segment 1 are displayed with a different Y-axis scale in S5 Fig.

Fig 3 summarizes the abundance of Ribo-seq and RNA-seq reads from protein-coding chloroplast genes in each of the four leaf segments. To display the low values from Segment 1, they are replotted with a smaller Y-axis scale in S5 Fig. The abundance of mRNA from genes in the same transcription unit (Fig 3A and S5A Fig, bracketed arrows) is typically similar, but the protein output of co-transcribed genes varies considerably. Translational efficiency (translational output /mRNA abundance) varies widely among genes (Fig 3A and S5A Fig, bottom). The *atpH* mRNA is the most efficiently translated of any chloroplast mRNA at all four developmental stages, surpassing even *psbA*, whose product is the most rapidly synthesized protein in photosynthetic tissues [26]. Prodigious *psbA* expression results from very high mRNA abundance in combination with a translational efficiency that is comparable to that of other photosystem genes.

**Fig 4:**
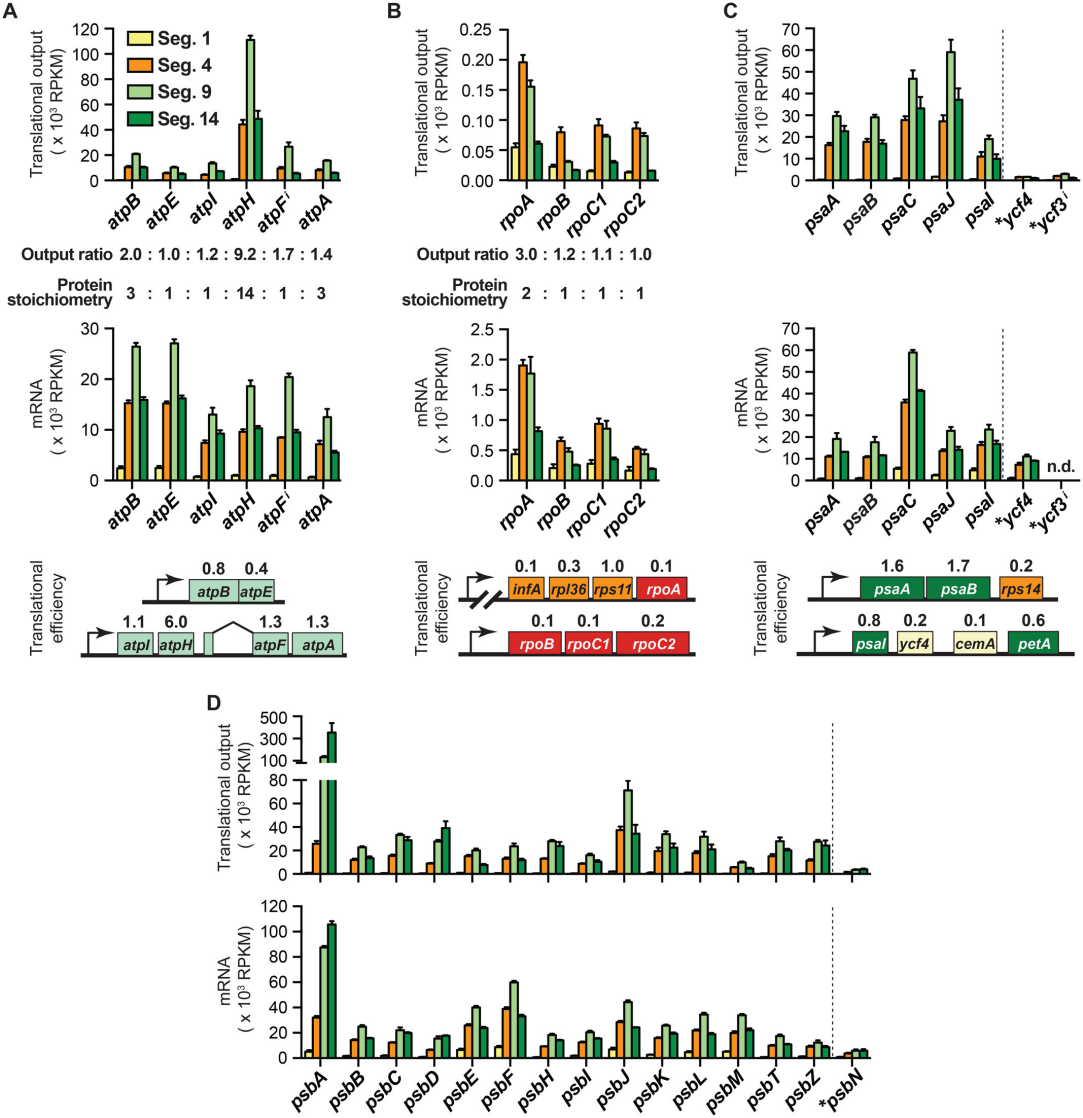
Comparison of protein stoichiometry, translational output and mRNA abundance for several complexes. Values represent the mean ± SEM from three replicates. RNA-seq data are not provided for *ycf3* due to uncertainty about the proportion of its transcripts that are fully spliced. Genes with introns are marked with superscript *i*. Genes encoding assembly factors are marked with asterisks. n.d. not determined. **(A)** Plastid genes encoding ATP synthase subunits. The Output Ratio values are averages of those in leaf segments 4, 9 and 14. The chloroplast transcription units encoding ATP synthase subunits are shown below, and are annotated with translational efficiencies from leaf segment 9. **(B)** Plastid genes encoding RNA polymerase subunits. The Output Ratio values are averages of those in leaf segments 1 and 4. The chloroplast transcription units encoding RNA polymerase subunits are shown below, and are annotated with translational efficiencies from leaf segment 9. Genes upstream of *rpoA* on the same polycistronic transcript are included to provide context. **(C)** Plastid genes encoding PSI subunits and assembly factors. Two of the chloroplast transcription units encoding PSI-related proteins are diagrammed below and annotated with translational efficiencies from leaf segment 9. **(D)** Plastid genes encoding PSII subunits and assembly factors.

When the data are grouped according to gene function, correlations between function and translational output become apparent (Fig 3B). For example, the translational output of genes encoding subunits of ribosomes and the NDH complex are consistently very low, whereas the translational output of genes encoding subunits of PSI, PSII, the ATP synthase, and the cytochrome *b*_*6*_*f* complex are consistently much higher. These trends mirror the abundance of these complexes as inferred from proteome data [27]. The data for complexes whose subunits are not found in a 1:1 ratio show further that translational output is tuned to subunit stoichiometry. For example, the chloroplast-encoded subunits of the ATP synthase (AtpA, AtpB, AtpE, AtpF, AtpH, AtpI) are found in a 3: 3: 1: 1: 14: 1 molar ratio in the complex [28, 29]. The translational output of their genes mirrors this stoichiometry quite well, whereas mRNA abundance does not (Fig 4A). These genes are distributed between two transcription units (Fig 4A). A single mRNA encodes AtpB and AtpE, whose rates of synthesis are tuned via differences in translational efficiency. The *atpI-atpH-atpF-atpA* primary transcript is processed to yield various smaller isoforms [30] but the abundance of RNA from each gene is nonetheless quite similar (Fig 4A). The translational output of the *atpH* gene is boosted relative to that of its neighbors primarily through exceptionally high translational efficiency (Fig 4A bottom). In a second example, the unequal stoichiometry of subunits of the plastid-encoded RNA polymerase (PEP) (2 RpoA:1 RpoB:1 RpoC1:1 RpoC2) [5] is mirrored by the relative translational output of the corresponding genes (Fig 4B). In this case, however, tuning occurs primarily at the level of mRNA accumulation.

The plastid-encoded subunits of PSI, PSII, the cytochrome *b*_*6*_*f* complex, the NDH complex, and chloroplast ribosomes are found in equal numbers in their respective complex. Genes encoding subunits of each of these complexes are distributed across multiple transcription units, many of which also encode subunits of other complexes. This gene organization sometimes results in considerable disparity in mRNA level among subunits of the same complex (Fig 3B bottom). In general, such differences are buffered by opposing changes in translational efficiency, such that translational outputs more closely reflect protein stoichiometry than does mRNA abundance (see, for example, the NDH complex in S6B Fig). In the case of PSI (Fig 4C), the structural genes (*psaA*, *psaB*, *psaC*, *psaJ*, *psaI*) exhibit an approximately three-fold range of translational output, but all of these genes vastly out produce two genes encoding PSI assembly factors (*ycf3* and *ycf4)* [31–33]. The *psaI* and *ycf4* genes are adjacent in the same polycistronic transcription unit (Fig 4C bottom), and their difference in translational output is programmed primarily by a difference in translational efficiency. The translational output of *psbN*, which encodes a PSII assembly factor [34], is likewise much less than that of structural genes for PSII (Fig 4D). Taken together, this body of data shows that the tuning of translational output to protein stoichiometries is accomplished via trade-offs between mRNA level and translational efficiency, with this balance differing from one gene to the next. Where mRNA abundance closely matches protein stoichiometry, differences in translational efficiency make only a small contribution (as observed for *rpoA*, *rpoB*, *rpoC1* and *rpoC2*). Where mRNAs are severely out of balance with protein stoichiometry, differences in translational efficiency compensate.

The translational output of PSII structural genes is well matched, with the notable exception of *psbA* (Fig 4D), whose output vastly exceeds that of other genes in photosynthetic leaf segments (segments 9 and 14). This behavior is consistent with the known properties of the *psbA* gene product, whose damage and rapid turnover during active photosynthesis is compensated by a high rate of synthesis to support PSII repair [26]. Setting *psbA* aside, the relative translational outputs of other genes only approximate the stoichiometries of their products: several-fold differences between relative output and stoichiometry are common among subunits of a particular complex, suggesting that proteolysis of unassembled subunits serves to fine-tune protein stoichiometries. It is also possible that the calculated translational outputs do not perfectly reflect rates of protein synthesis due to differences in translation elongation rates among mRNAs. That said, instances in which translational outputs are particularly discordant among subunits of the same complex are worthy of note, as this may reflect physiologically relevant behaviors. For example, the translational output of *ndhK* is balanced with other *ndh* genes in non-photosynthetic leaf segments but *ndhK* substantially out produces the other *ndh* genes in mature chloroplasts (S6B Fig). This behavior is reminiscent of *psbA*, and suggests that NdhK may be damaged and replaced during active photosynthesis.

### Developmental dynamics of the chloroplast transcriptome and translatome

**Fig 5:**
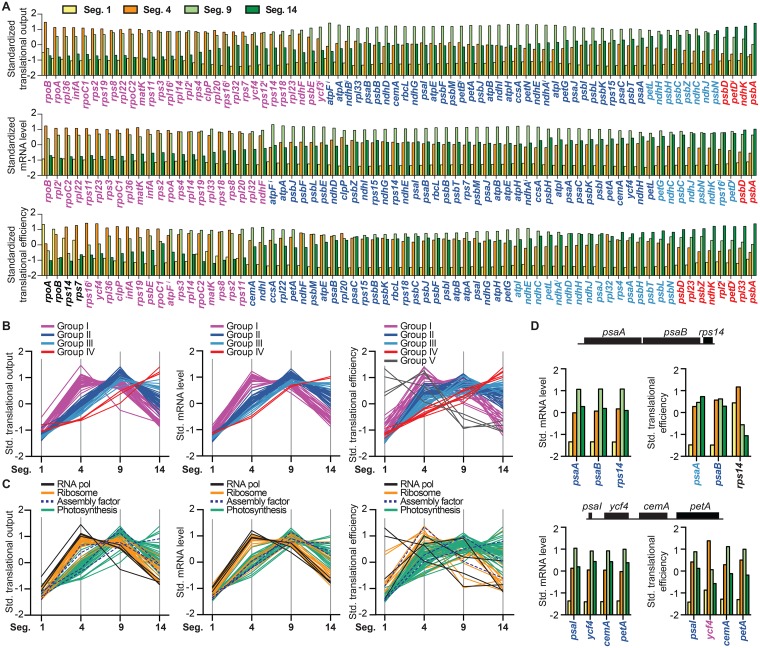
Dynamics of chloroplast gene expression during the proplastid to chloroplast transition. Values for each gene in the four leaf segments were standardized to have a mean of 0 and a standard deviation of 1. Standardized values for translational output, mRNA abundance, and translational efficiency were used for hierarchical clustering. Genes are color-coded according to the cluster they reside in, as defined by the clustograms shown in S7 Fig. Values represent the mean from three replicates. **(A**) Genes are ranked based on the clustograms shown in S7 Fig. Genes for which mRNA levels are uncertain (as explained above) are not included in the plots of mRNA level and translational efficiency. Intron-containing genes are marked with superscript *i*. **(B)** Developmental dynamics of each cluster derived from the data for translational output, mRNA abundance, or translational efficiency. Each line represents data from one gene. **(C)** Developmental dynamics of translational output, mRNA level, and translational efficiency color-coded according to gene function. Each line represents data from one gene. **(D)** Excerpts of the data for two transcription units encoding proteins that function in both photosynthesis (*psaI*, *petA*, *psaA*, *psaB)* and biogenesis of the photosynthetic apparatus (*ycf4*, *rps14)*.

To explore the dynamics of chloroplast gene expression during the proplastid to chloroplast transition, we calculated standardized values for translational output, mRNA abundance and translational efficiency such that developmental shifts can be compared despite large differences in signal magnitude. This analysis shows that the developmental dynamics of translational output varies widely among genes (Fig 5A top). The standardized values were used as the input for hierarchical clustering, which produced four clusters from the translational output data, four from the mRNA data, and five from the translational efficiency data (Fig 5B, S7 Fig). The genes in each cluster are identified by color in Fig 5A. Although the transitions between clusters are not marked by obvious distinctions, the distinct trends defining each cluster are clear in the plots in Fig 5B. Genes whose translational output and mRNA abundance peak early in development (segment 4) generally encode components of the chloroplast gene expression machinery (*rpl*, *rps*, *rpo*, *matK*) (Fig 5A and 5C). Most genes encoding components of the photosynthetic apparatus (*psb*, *psa*, *atp*, *pet* genes) have peak mRNA and translational output in young chloroplasts (segment 9). A handful of photosynthesis genes either maintain or increase translational output and mRNA in mature chloroplasts (segment 14) (Fig 5A and 5C).

There is considerable similarity among the clusters produced from the translational output and mRNA data (Fig 5A and 5B), implying that programmed changes in mRNA abundance underlie the majority of developmental shifts in translational output. However, changes in translational efficiency also influence the developmental shifts in translational output (Fig 5A bottom). In general, ORFs encoding proteins involved in photosynthesis are more efficiently translated later in development and those encoding gene expression factors are more efficiently translated early in development, albeit with numerous exceptions (Fig 5A bottom, 5C right). Transcription units that encode both photosynthesis and gene expression factors provide revealing examples of distinct translational dynamics. In the *psaA-psaB-rps14* transcription unit, for example, *rps14* is found in a translational output cluster with other genes involved in gene expression, whereas *psaA* and *psaB* reside in a translational output cluster with other photosynthesis genes (Fig 5A top). This results from distinct developmental shifts in translational efficiency: the *rps14* ORF is translated more efficiently early in development whereas *psaA* and *psaB* are more efficiently translated later in development (Fig 5D). The *psaI-ycf4-cemA-petA* transcription unit provides a second example. The translational output of *psaI*, *cemA*, and *petA* show similar developmental dynamics, but *ycf4* clusters with different genes due to more efficient translation earlier in development (Fig 5D). Again, these distinct patterns correlate with function, as *psaI* and *petA* encode components of the photosynthetic apparatus, whereas *ycf4* encodes an assembly factor for PSI [31, 32].

Many polycistronic RNAs in chloroplasts are processed to smaller isoforms. Although the impact of processing on translational efficiencies remains unclear [35, 36], it is plausible that programmed changes in the accumulation of processed isoforms could uncouple the expression of cotranscribed genes during development. To address this possibility, we used RNA gel blot hybridization to analyze transcripts from two transcription units that include genes whose translational efficiencies exhibit distinct developmental dynamics: *psaI-ycf4-cemA-petA* and *psaA-psaB-rps14* transcription units (S8 Fig). Processed *rps14*-specific transcripts accumulate preferentially in immature chloroplasts (segment 4), correlating with the stage at which *rps14* is most efficiently translated. Analogously, a monocistronic *psaI* isoform accumulates preferentially in segments 4 and 9 where *psaI* is most efficiently translated. Various cause and effect relationships may underlie these correlations, as is discussed below.

### Differential gene expression in bundle sheath and mesophyll chloroplasts

**Fig 6:**
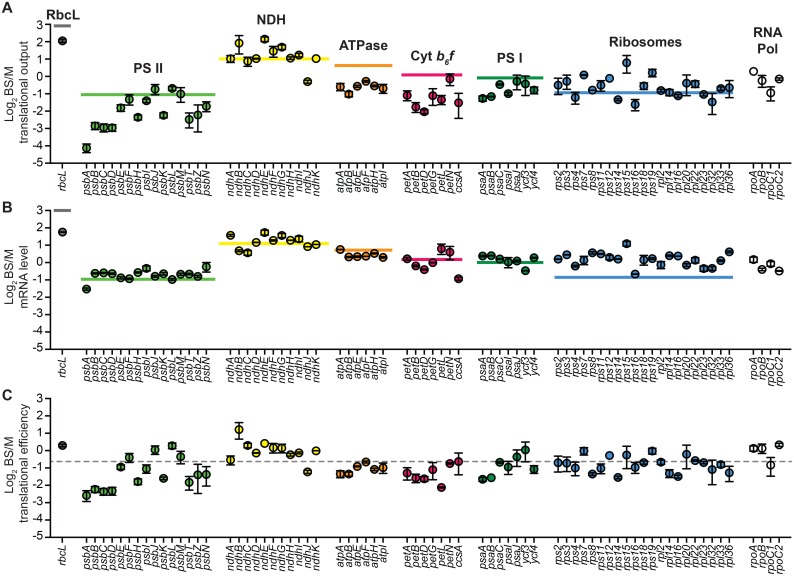
Differential gene expression in bundle sheath (BS) and mesophyll (M) chloroplasts. Values are the mean ± SEM from two replicates. The horizontal lines show the average BS/M ratios for subunits of each complex determined from quantitative proteome data [27]. Immunoblots illustrating the abundance of marker proteins in these samples are shown in Fig 1C. **(A)** Relative translational output in BS versus M fractions. **(B)** Relative mRNA abundance in BS versus M fractions. **(C)** Relative translational efficiency in BS versus M fractions. The dashed line marks the average ratio of translational efficiencies in BS versus M fractions.

In maize and other C4 plants, photosynthesis is partitioned between mesophyll (M) and bundle sheath (BS) cells. Three protein complexes that include plastid-encoded subunits accumulate differentially in the two cell types: Rubisco and the NDH complex are enriched in BS cells whereas PSII is enriched in M cells [2, 14]. Differential accumulation of several chloroplast mRNAs in the two cell types has been reported [37–41], but a comprehensive comparison of chloroplast gene expression in BS and M cells has been lacking. To address this issue we performed RNA-seq and Ribo-seq analyses of BS- and M- enriched leaf fractions. The translational output of genes encoding subunits of Rubisco, PSII, and the NDH complex (Fig 6A) correlated well with the relative abundance of subunits of these complexes in the same sample preparations (Fig 1C), and with quantitative proteome data [2]. Cell-type specific differences in mRNA accumulation (Fig 6B) can account for many of the differences in translational output (Fig 6A), indicating that differences in transcription and/or RNA stability make a strong contribution to preferential gene expression in one cell type or the other. However, the data suggest that differences in translational efficiency contribute in certain instances (Fig 6C). Four genes encoding PSII core subunits (*psbA*, *psbB*, *psbC*, *psbD)* provide the most compelling examples, as their translational output is considerably more biased toward M cells than are their mRNA levels.

### Translation of edited and unedited RNAs

**Fig 7:**
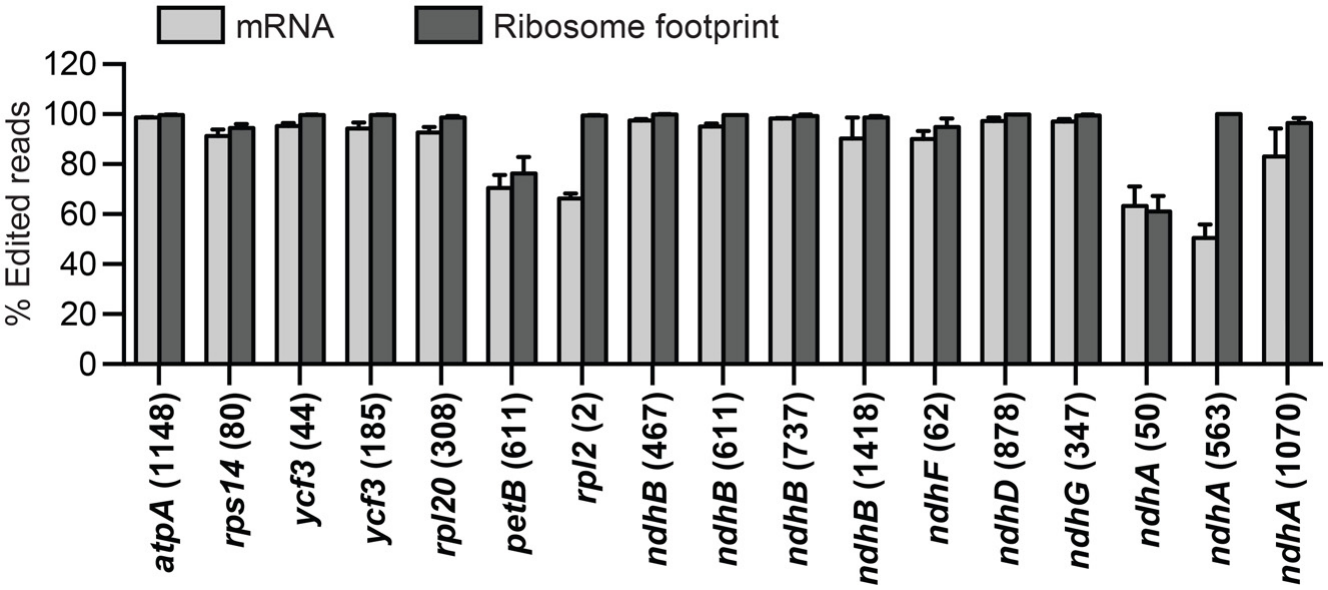
Abundance of ribosome footprints representing edited and unedited mRNA positions. Data are shown for the subset of editing sites that were represented by at least 100 reads in both the RNA-seq and Ribo-seq data in at least two different samples from the leaf gradient analysis. Values are the mean ± SEM.

Organellar RNAs in land plants are often modified by an editing process that converts specific cytidine residues to uridine [42, 43]. Some sites are inefficiently edited, which raises the question of whether the translation machinery discriminates between edited and unedited RNAs. The protein products of several unedited mitochondrial RNAs have been detected in plants [44, 45]. We used our Ribo-seq and RNA-seq data to examine this issue for chloroplast RNAs. Fig 7 summarizes the data for those sites of editing that are represented by at least 100 reads in both the Ribo-seq and RNA-seq data in at least two replicates (17 of the 28 edited sites in the maize chloroplast transcriptome). In general, the percent editing was similar in the RNA-seq and Ribo-seq data, implying little discrimination between edited and unedited RNAs by the translation machinery. There were, however, two major exceptions: *rpl2* (nt 2) and *ndhA* (nt 563). In these cases a large fraction of the RNA-seq reads came from unedited RNA, whereas virtually all of the Ribo-seq reads came from edited sites. These two sites have unusual features that can account for the preferential translation of the edited RNAs. Editing at the *ndhA* site is linked to the splicing of the group II intron in the *ndhA* pre-mRNA: the site is not edited in unspliced transcripts and it is fully edited in spliced transcripts [46–48]. Failure to edit unspliced RNA is presumably due to the position of the intron between the edited site and the cis-element that specifies it. Translation that initiates on unspliced *ndhA* RNA would terminate at an in-frame stop codon within the intron. Thus, exon 2 is translated only from spliced RNAs, and these are 100% edited. In the case of *rpl2*, the editing event creates an AUG start codon from an ACG precursor; this is the only editing event in maize chloroplasts that creates a canonical start codon. Although it has been reported that ACG can function as a start codon in chloroplasts [49, 50], our data show that this particular ACG is strongly discriminated against by initiating ribosomes.

The fact that the Ribo-seq data show the expected strong bias toward edited *rpl2* and *ndhA*(563) instills confidence that valid conclusions can be made from our data for other edited sites. Approximately 40% of the *petB* and *ndhA*(nt 50) sequences are unedited in both the RNA-seq and Ribo-seq data, indicating that these unedited sequences give rise to a considerable fraction of the translational output of the corresponding genes. Editing of the *petB* site is essential for the function of its gene product (cytochrome *b*_*6*_) [51]. It seems likely that the product of this unedited RNA is either unstable or selected against during complex assembly, as has also been suggested for the products of two unedited transcripts in mitochondria [52, 53]. The remaining sites show almost complete editing in the RNA-seq data and, as expected, in the Ribo-seq data as well. That said, there is an overall trend toward less representation of unedited sequences in the Ribo-seq data than in the RNA-seq data. This may simply be a kinetic effect as would be expected if ribosome binding is slow in comparison to editing, such that ribosomes generally translate older (and therefore more highly edited) mRNAs.

## Discussion

Ribosome profiling has provided a wealth of new insights into translation and associated processes in a wide variety of organisms [54], but its application to questions in organellar biology is just beginning. The method has been used to analyze the effects of a disease-associated mutation in mitochondria [19], to define targets of nucleus-encoded translational activators in chloroplasts [15] and to characterize the cotranslational targeting of chloroplast-encoded proteins to the thylakoid membrane [36]. The results reported here provide the first comprehensive description of an organellar transcriptome and translatome in a developmental context. The data revealed dynamic changes in RNA abundance and translational efficiency during the differentiation of proplastids into chloroplasts, elucidated mechanisms that dictate the abundance of chloroplast-encoded proteins, clarified the relationship between RNA editing and translation, and provided new insights that suggest hypotheses to be explored in future studies.

### Tuning of protein synthesis to protein stoichiometry: Close but not quite

Ribosome profiling data from bacteria revealed a striking correspondence between the stoichiometry of subunits of multisubunit complexes and their relative rates of synthesis [9, 10]. Our results show that the relative translational outputs of chloroplast genes likewise approximate the relative abundance of the gene products. This tuning is apparent when comparing sets of genes encoding different complexes (e.g. compare genes encoding the low abundance NDH complex to genes encoding the highly abundant PSI and PSII complexes) (Fig 3B), and when comparing genes encoding subunits of the same complex (e.g. the PEP RNA polymerase and the ATP synthase) (Fig 4A and 4B).

Our calculations of translational output rest on the assumption that the rate of translation elongation on all mRNAs is similar under any particular condition. This same assumption produced remarkable concordance between protein stoichiometry and inferred translational output in bacteria [9, 10]. Although our results show a clear trend toward “proportional synthesis”, they also suggest that the tuning of protein output to stoichiometry is less precise in chloroplasts than it is in bacteria. Subunits of photosynthetic complexes are subject to proteolysis when their assembly is disrupted [55], and a similar (albeit wasteful) mechanism could contribute to balancing stoichiometries when proteins are synthesized in excess under normal conditions. That said, instances in which inferred translational outputs are particularly incongruent with protein stoichiometries may reflect physiologically informative behaviors. The most prominent examples of “over-produced” proteins in our data are PsbA and PsbJ in PSII, PsaC and PsaJ in PSI, NdhK in the NDH complex, Rps14 in ribosomes, and PetD, PetL and PetN in the cytochrome *b*_*6*_*f* complex (Fig 4 and S6 Fig). Disproportionate synthesis of PsbA is well known, and compensates for its damage and proteolysis during photosynthesis [26]. The other proteins suggested by our data to be produced in excess may likewise be subject to more rapid turnover than their partners in the assembled complex. A proteomic study in barley demonstrated that subunits of each photosynthetic complex generally turn over at similar rates [56], but data for these particular proteins were not reported.

Interestingly, the inferred rates of synthesis of PsbA, PetD, and NdhK are well matched to those of their partner subunits early in development, but outpace those of their partners in mature chloroplasts (Fig 4D,S6 Fig). This feature of *psbA* expression coincides with the need to replace its gene product, D1, following photo-induced damage and proteolysis [26]. By extension, the developmental dynamics of *petD* and *ndhK* expression suggest that their gene products may turn over more rapidly than their partners as a consequence of photosynthetic activity.

### Varying contributions of mRNA abundance and translational efficiency to the tuning of protein output

In bacteria, proportional synthesis of subunits within a complex is achieved largely through the tuning of translational efficiencies among ORFs on the same mRNA [9, 10]. In chloroplasts, genes encoding subunits of the same complex are generally distributed among multiple transcription units [4] and RNA segments within a transcription unit often accumulate to different levels [8]. It is interesting to consider how this shift in the gene expression landscape is reflected in the mechanisms that balance protein output among genes. In the case of the four genes encoding the PEP RNA polymerase, relative translational outputs closely match the 2:1:1:1 protein stoichiometry, and this is programmed primarily at the level of mRNA abundance (Fig 4B). By contrast, widely varying translational efficiencies are superimposed on small variations in mRNA abundance to tune translational output to protein stoichiometry in the ATP synthase complex (Fig 4A). Genes for ribosomal proteins are distributed among ten transcription units, several of which also encode proteins involved in photosynthesis (see Fig 3A). For example, *rps14* is cotranscribed with genes encoding the reaction center proteins of PSI (*psaA/psaB*), and translational outputs within this transcription unit are balanced by large differences in translational efficiency (Fig 4C). Similarly, the *psaI* transcription unit encodes subunits of the abundant PSI and cytochrome *b*_*6*_*f* complexes, a low abundance PSI assembly factor (Ycf4) and a protein of unknown function (CemA); large differences in translational efficiency adjust the translational outputs to meet these different needs (Fig 4C bottom). For complexes harboring plastid-encoded subunits in equal stoichiometries (ribosomes, NDH, PSI, PSII, cytochrome *b*_*6*_*f*), compensating differences in translational efficiency generally buffer differences in mRNA level. Taken together, these results imply that mRNA abundance and translational efficiencies have coevolved in chloroplasts to produce proteins in close to the optimal amounts. In some instances, mRNA levels are sharply out of balance with protein stoichiometries, in which case differential translational efficiencies compensate. In other instances, mRNA levels approximate protein stoichiometries, and translational efficiencies are similar. These observations further suggest that for most genes in maize chloroplasts, mRNA levels and translational efficiencies are poised such that they limit the rate of protein synthesis to a similar extent. This view is further supported by the developmental dynamics discussed below.

In Chlamydomonas chloroplasts, synthesis of subunits within the same photosynthetic complex is coordinated through assembly-dependent auto-regulatory mechanisms [57]. By contrast, current data for angiosperm chloroplasts suggest that translational efficiencies are generally independent of the assembly status of the gene products [15, 58]. It seems likely that translational efficiencies are dictated by the interplay between the sequence and structure of RNA proximal to start codons and the proteins that bind this region. Translation initiation in chloroplasts sometimes involves a Shine-Dalgarno interaction and is facilitated by an unstructured translation initiation region [6, 59]. Additionally, the translation of some chloroplast ORFs requires the participation of gene-specific translation activators [15, 60–75]. Such proteins provide a means for tuning protein synthesis within and between transcription units. The *atpH* ORF and its nucleus-encoded translational activator PPR10 exemplify this mechanism. The exceptionally high translational efficiency of *atpH* (Fig 3A) boosts its translational output to match the high stoichiometry of AtpH in the ATP synthase complex (Fig 4A); this high translational efficiency requires the binding of PPR10 adjacent to the *atpH* ribosome binding site, an interaction that prevents the formation of inhibitory RNA structures involving the translation initiation region [15, 30, 62].

### Developmental dynamics of chloroplast gene expression

Our results provide a comprehensive view of the dynamics of chloroplast mRNA abundance and translation during the proplastid to chloroplast transition. The majority of genes involved in chloroplast gene expression exhibit peak mRNA abundance and translational output in developing chloroplasts (segment 4) whereas the majority of genes encoding subunits of the photosynthetic apparatus exhibit peak mRNA abundance and translational output in young chloroplasts (segment 9) (Fig 5C). That said, even in proplastids (segment 1), genes involved in photosynthesis are generally represented by more mRNA and a higher translational output than are those involved in chloroplast gene expression (S5 Fig). Our data show that programmed changes in translational efficiency combine with changes in mRNA abundance to produce developmental shifts in translational output (Fig 5A). In general, translational efficiency is lowest at the leaf base, reflecting the low ribosome content in proplastids. The translational efficiency of most ORFs peaks in young chloroplasts (segment 9). In this context, it is intriguing that one subset of genes exhibit peak translational efficiency in the basal leaf segments (Fig 5A, bottom; Fig 5C, right), whereas another subset increases in translational efficiency right out to the leaf tip (Fig 5A and 5C). The former group is strongly enriched for “biogenesis” genes (RNA polymerase, ribosomes, assembly factors), and the latter for photosynthesis genes. Possible mechanisms underlying these distinct “translational regulons” are discussed below.

A study in Chlamydomonas showed that changes in chloroplast mRNA abundance are not reflected by corresponding changes in rates of protein synthesis, leading to the conclusion that translation is the primary rate-limiting step [76]. The data presented here suggest that this is not the case in maize chloroplasts. The developmental shifts in mRNA abundance were largely mirrored by shifts in translational output (Fig 5A and 5B), implying that mRNA abundance has considerable impact on the output of most chloroplast genes in maize. Likewise, chloroplast DNA copy number limits gene expression in developing maize chloroplasts [77] but does not limit gene expression in Chlamydomonas chloroplasts [76]. It is perhaps unsurprising that mechanisms of gene regulation have diverged in the chloroplasts of vascular plants and single-celled algae, given their very different developmental and ecological contexts.

### Mechanisms underlying developmental shifts in the chloroplast transcriptome and translatome

Our data revealed a strong correlation between gene function and the developmental dynamics of mRNA abundance (Fig 5C middle): mRNAs encoding proteins involved in gene expression generally peak in abundance earlier in development than do those encoding components of the photosynthetic apparatus. This finding was foreshadowed by analyses of several chloroplast mRNAs during leaf development in barley and Arabidopsis [78–80]. Land plant chloroplasts harbor two types of RNA polymerase, a single-subunit nucleus-encoded polymerase (NEP) and a bacterial-type plastid-encoded polymerase (PEP) [5]. The ratio of NEP to PEP drops precipitously during chloroplast development, and this likely makes a large contribution to the changes in chloroplast mRNA pools [5, 78, 80, 81]. There is evidence that NEP plays an especially important role in the transcription of “house keeping” genes, and PEP in the transcription of photosynthesis genes [5]; however, most chloroplast genes can be transcribed by both NEP and PEP [82], and the degree to which each polymerase contributes to the transcription of each gene during the course of chloroplast development remains unknown. Chloroplasts harbor several nucleus-encoded sigma factors that target PEP to distinct promoters [83], and these provide an additional means to tune transcription rates in a developmental context.

Changes in RNA stability combine with changes in transcription to modulate mRNA pools during chloroplast development [78, 80, 81, 84, 85]. Determinants of chloroplast mRNA stability include various ribonucleases, RNA structure, ribosome occupancy, and proteins that protect RNAs from nuclease attack [8]. Most mRNA termini in chloroplasts are protected by helical repeat RNA binding proteins that provide a steric blockade to exoribonucleases [8, 61, 62, 86]. The majority of such proteins belong to the pentatricopeptide repeat (PPR) family, a large family of sequence-specific RNA binding proteins that influence virtually every post-transcriptional step in gene expression in mitochondria and chloroplasts [87]. In addition, chloroplasts harbor abundant hnRNP-like proteins, and these have been shown to impact the stability of several chloroplast mRNAs [88, 89]. Programmed changes in the abundance and/or activities of PPR and hnRNP-like proteins might contribute to the shifting mRNA pools during the proplastid to chloroplast transition.

Changes in translational efficiency superimpose on changes in mRNA abundance to modulate the output of plastid genes during the transformation of proplastids into chloroplasts. ORFs encoding proteins involved in photosynthesis generally exhibit maximal translational efficiency in young or mature chloroplasts (segments 9 and 14), whereas those that function in gene expression generally peak in translational efficiency earlier in development (Fig 5C right). Furthermore, our data suggest that mRNAs encoding PSII reaction center proteins are translated with higher efficiency in mesophyll chloroplasts than in bundle sheath chloroplasts (Fig 6C). It will be interesting to explore the mechanisms that underlie these differential effects on translational efficiency. Some possibilities include shifts in stromal pH, Mg^++^, or the polymerase generating the mRNA (NEP versus PEP), which might impact the formation of RNA structures at specific ribosome binding sites. Programmed changes in the activities of nucleus-encoded gene-specific translational activators could modulate translational efficiencies in a developmental context. Most such proteins in land plant chloroplasts are PPR (or PPR-like) proteins, and several of these also stabilize processed mRNAs with a 5’ end at the 5’ boundary of their binding site [15, 30, 60–62, 64–67, 90–93]. Indeed, many polycistronic transcripts in chloroplasts are processed to smaller isoforms whose ends are defined and stabilized by PPR-like proteins [7, 8, 86]. The impact of this type of RNA processing on translational efficiencies *in vivo* remains unclear. The removal of upstream ORFs is not required for the translation of several ORFs that are found on processed RNAs with a proximal 5’-terminus [35, 36]. Some proteins have dual translation activation and RNA processing/stabilization functions, implying that the two activities are coupled [15, 30, 60–62, 65, 66, 91–93]; however, the translation activation and RNA processing/ stabilization effects of such proteins could be independent consequences of their binding upstream of an ORF [62, 86]. We showed here that there is a correlation between the accumulation of processed RNA isoforms and changes in relative translational efficiencies in two polycistronic transcription units (S8 Fig). Deciphering the cause and effect relationships underling these correlations presents a challenge for the future.

The data presented here lead to numerous new questions for future exploration. Is the synthesis of nucleus-encoded subunits of photosynthetic complexes tuned to that of their chloroplast-encoded partners? What is the mechanistic basis for the preferential translation of some mRNAs in developing chloroplasts and others in photosynthetic chloroplasts? To what extent do environmental inputs such as light and temperature modify the developmental dynamics of chloroplast mRNA abundance and translation? The use of ribosome profiling can be anticipated to accelerate progress in addressing these and many other long-standing questions relating to the biology of organelles.

## Materials and Methods

### Plant material

For the developmental analysis, *Zea mays* (inbred line B73) was grown under diurnal cycles for 9 days and harvested as described [12]. Leaf sections from twelve plants were pooled for each of three replicates; each pool contained between 0.15 g and 0.3 g tissue. Plants used to prepare mesophyll and bundle sheath fractions were grown similarly, except the light was set at 300 μmol·m^-2^·s^-1^ and the tissues were harvested 13 days after planting, 2 hours into the light cycle. The apical one-third of leaf two and three were pooled from fifteen seedlings for each replicate, and the bundle sheath and mesophyll-enriched fractions were obtained with a rapid mechanical procedure. The tissue was cut into ~ 1 cm-sections, placed in a pre-chilled mortar and pestle, and lightly ground for 2 min in 5 ml of ice-cold modified polysome extraction buffer lacking detergents (0.2 M sucrose, 0.2 M KCl, 50 mM Tris-acetate, pH 8.0, 15 mM MgCl_2_, 20 mM 2-mercaptoethanol, 2 μg/ml pepstatin A, 2 μg/ml leupeptin, 2 mM phenylmethanesulfonyl fluoride, 100 μg/ml chloramphenicol, 100 μg/ml cycloheximide). The material that was released into solution constituted the mesophyll cell-enriched fraction. A portion of this was frozen in liquid N_2_ for RNA isolation, and the remainder was stored on ice while bundle sheath strands were purified from the tissue remaining in the mortar. The tissue was subject to four additional rounds of light grinding (2 min), each time in a fresh aliquot of 5-ml modified polysome extraction buffer. The light green fibers remaining constituted the bundle sheath enriched fraction; these cells were broken by hard grinding in 5 ml of modified polysome extraction buffer. A portion of this material was flash frozen for future RNA isolation and the remainder was used immediately for ribosome footprint isolation. Polyoxyethylene (10) tridecyl ether and Triton X-100 were added to the mesophyll and bundle sheath fractions retained for ribosome profiling (final concentrations of 2% and 1%, respectively), and the material was filtered through glass wool. The isolation of ribosome footprints and total RNA were performed as described below.

### Preparation of ribosome footprints

Ribosome footprints were prepared using a protocol similar to that described in [15], but with two key modifications: (i) RNAse I rather than micrococcal nuclease was used to generate monosomes, and (ii) the centrifugation time used to pellet ribosomes through the sucrose cushion was shortened to reduce contamination by other RNPs. Tissues were pulverized in liquid N_2_ with a mortar and pestle, and thawed in 5 ml of polysome extraction buffer (0.2 M sucrose, 0.2 M KCl, 50 mM Tris-acetate, pH 8.0, 15 mM MgCl_2_, 20 mM 2-mercaptoethanol, 2% polyoxyethylene (10) tridecyl ether, 1% Triton X-100, 100 μg/ml chloramphenicol, 100 μg/ml cycloheximide). A 2.4-ml aliquot was removed and frozen in liquid N_2_ for total RNA isolation. The remaining suspension was filtered through glass wool and centrifuged at 15,000xg for 10 min. The supernatant was digested with 3,500 units of RNAse I (Ambion) at 23°C for 30 min. 2.5 ml lysate was layered on a 2 ml sucrose cushion (1 M sucrose, 0.1 M KCl, 40 mM Tris-acetate, pH 8.0, 15 mM MgCl_2_, 10 mM 2-mercaptoethanol, 100 μg/ml chloramphenicol, and 100 μg/ml cycloheximide) in a 16 x 76 mm tube and centrifuged in a Type 80 Ti rotor for 1.5 h at 55,000 rpm. The pellet was dissolved in 0.7 mL of ribosome dissociation buffer (10 mM Tris-Cl, pH 8.0, 10 mM EDTA, 5 mM EGTA, 100 mM NaCl, 1% SDS). RNA was isolated with Tri reagent (Molecular Research Center). RNAs between ~20 and ~35 nt were purified on a denaturing polyacrylamide gel, eluted, extracted with phenol/chloroform, precipitated with ethanol, and suspended in water. We have subsequently modified our protocol to purify RNAs between 20 and 40 nt; this results in a small shift in the size distribution of the reads (S1B Fig).

### Preparation of sequencing libraries

The ribosome footprint preparation was treated with T4 polynucleotide kinase. Twenty ng of the kinased RNA was converted to a sequencing library using the NEXTflex Small RNA Sequencing Kit v2 (Bioo Scientific), which minimizes ligation bias by introducing four randomized bases at the 3’ ends of the adapters [17]. rRNA fragments were depleted by subtractive hybridization after first-strand cDNA synthesis, using 54 biotinylated DNA oligonucleotides corresponding to the most abundant rRNA fragments detected in pilot experiments (see S1 Table). 10 μl of the oligonucleotide mixture (concentrations as in S1 Table) was added to 40-μl of the first-strand synthesis reaction and heated to 95°C for 2 min. A 50-μl aliquot of pre-warmed 2X hybridization buffer (10 mM Tris-Cl pH 7.5, 1 mM EDTA, 2 M NaCl) was added and incubated at 55°C for 30 min. The solution was transferred to a new tube containing 1 mg of prewashed Dynabeads M-270 Streptavidin (Invitrogen) and incubated at room temperature for 15 min with frequent agitation. The tube was placed on a magnet for 5 min and the supernatant was collected and desalted using Sephadex G-25 Fine (GE Healthcare). The sample was concentrated to 18 μl and used as input for the PCR amplification step in the library construction protocol. After 14 cycles, PCR products were separated by electrophoresis through a 5% polyacrylamide gel and a gel slice corresponding to DNA fragments between markers at 147 and 180 bp (representing insert sizes of 20–53 bp) was excised. The DNA was eluted overnight, phenol/chloroform extracted, precipitated with ethanol, suspended in water, and stored at -20°C.

For RNA-seq, rRNA was depleted from the RNA samples using the Ribo-Zero rRNA Removal Kit (Plant Leaf) (Epicentre). One hundred ng of the rRNA-depleted RNA was used for library construction using the NEXTflex Rapid Directional qRNA-Seq Kit (Bioo Scientific) according the manufacturer’s instructions. The adapters provided with the kit include 8-nt molecular labels that were used during data processing to remove PCR bias. The libraries were combined and sequenced using a HiSeq 2500 or NextSeq 500 instrument (Illumina). The read lengths were 50 or 75 nt for Ribo-seq and 75 nt for mRNA-seq.

### Sequence read processing, alignment, and analysis

Adapter sequences were trimmed using cutadapt [94]. Ribo-seq reads between 18 and 40 nt were used as input for alignments. Alignments were performed using Bowtie 2 with default parameters [95], which permits up to 2 mismatches, thereby allowing edited sequences to align. Reads were aligned to the following gene sets, with unaligned reads from each step used as input for the next round of alignment: (i) chloroplast tRNA and rRNA; (ii) chloroplast genome; (iii) mitochondrial tRNA and rRNA; (iv) mitochondrial genome (B73 AGP v3); (v) nuclear tRNA and rRNA; nuclear genome (B73 AGP v3).

Maize genome annotation 6a (phytozome.jgi.doe.gov) was reduced to the gene set annotated in 5b+ (60,211 transcripts) (gramene.org). For metagene analysis, all coding sequence (CDS) coordinates from all transcript variants were combined to make a union CDS coordinate. Custom Perl scripts extracted mapping information using SAMtools [96] and analyzed mapped reads as follows. The distribution of ribosome footprint lengths and the RPKM for both the Ribo-seq and RNA-seq data were calculated based only on reads mapping to CDS regions. For RPKM calculations, we defined the total number of mapped reads as the number of reads mapping to nuclear CDSs. Translation efficiency was calculated from the division of ribosome footprint RPKM by RNA-seq RPKM.

Because unspliced RNAs constitute a substantial fraction of the RNA pool from intron-containing genes in chloroplasts, these genes require special treatment to infer the abundance of spliced (functional) mRNA. The fraction spliced at each intron was calculated in several ways. (i) RNA-seq reads were aligned to the chloroplast genome with splicing-aware software TopHat 2.0.11 [97]. The number of reads spanning each exon-exon junction (spliced) was divided by the sum of spliced (exon-exon) and unspliced (exon 1-intron or intron-exon 2) reads; (ii) RNA-seq reads were aligned with Bowtie2 to a reference gene set that included both spliced and unspliced forms (100-nt on each side of each junction). The fraction of spliced RNA was calculated as for method (i); (iii) RNA-seq reads were aligned with TopHat to the genome and the spliced fraction was calculated from (exon RPKM—intron RPKM)/exon RPKM. Values calculated by each method are provided in S3 Table. Summary plots report mRNA abundance and translational efficiencies only for genes for which all of these methods gave similar results. We cannot confidently infer the amount of fully spliced RNAs from genes with two introns (*ycf3* and *rps12)*, so these are also excluded.

Hierarchical clustering was performed using the Bioinformatic Toolbox of MATLAB software (Mathworks) using standardized values as input: values from the four leaf segments for each gene were standardized to have a mean of 0 and a standard deviation of 1 such that developmental shifts can be compared among genes despite differences in signal magnitude. Hierarchical clustering was performed using Pearson correlation coefficient values and unweighted average distance.

### Antibodies

Antibodies to AtpB, D1 and PetD were raised by our group and have been described previously [30]. Antibodies to Atp6, NdhH, PPDK, and Rpl2 were generously provided by Christine Chase (University of Florida), Tsuyoshi Endo (Kyoto University), Kazuko Aoyagi (UC Berkeley), and Alap Subramanian (University of Arizona), respectively. Antibodies to PEPC, malic enzyme, and RbcL were generous gifts of William Taylor (University of California, Berkeley).

### Accession numbers

Illumina read sequences were deposited at the NCBI Sequence Read Archive with accession number SRP070787. Alignments of reads to the maize chloroplast genome used Genbank accession X86563.

## Supporting Information

S1 Fig Optimized ribosome purification reduces contamination by non-ribosomal ribonucleoprotein particles (RNPs). **(A)** Immunoblots showing that a marker for chloroplast ribosomes (Rpl2) was highly enriched in the pellet after sedimentation of nuclease-treated extract through a sucrose cushion, whereas a subunit of a ~600 kDa group II intron RNP (CFM2) [16] remained in the supernatant. An equal proportion of the starting material, the supernatant above the sucrose cushion, and the pellet fraction was analyzed. **(B)** Comparison of size distribution of chloroplast ribosome footprints resulting from two different size selection strategies. The experiments in this study used gel purified RNA fragments between approximately 20 and 35-nt (green). A pilot experiment used gel-purified RNA fragments between approximately 20 and 40-nt (blue). The size distribution of the sequence reads was nonetheless similar.
(TIF)

S2 Fig Correlation of data among biological replicates. Pearson correlation coefficients for each sample pair combination were calculated using log_10_ of RPKM values for each protein-coding gene in the chloroplast genome. The correlation coefficients were used as the input for hierarchical clustering. The replicate number of each leaf segment sample is indicated after the hyphen. **(A)** Leaf segment data. **(B)** Bundle sheath (BS) and mesophyll (M) data.
(TIF)

S3 Fig Summary of read counts per chloroplast gene. The values displayed are the mean from replicate assays. The identities of genes with low read counts are indicated. The *petN* mRNA is under represented in the RNA-seq data due to its small size, which is below the cut-off used for library preparation. **(A)** Read counts/gene for leaf gradient samples. **(B)** Read counts/gene for bundle sheath (BS) and mesophyll (M) samples.
(TIF)

S4 Fig Characteristics of ribosome footprints in each compartment. The data plotted come from the twelve leaf gradient samples. **(A)** Three-nucleotide periodicity of P site position as a function of ribosome footprint length in the plastid, cytosol, and mitochondrion. Values shown are the mean ± SEM. The position of the P site in each footprint was inferred from the footprint size distributions at start/stop codons (Fig 2E and S4B-C). **(B)** Placement of cytosolic ribosome footprints with respect to the A and P sites of the ribosome based on reads aligning to start and stop codons. A diagram of the placement of 31-nucleotide cytosolic ribosome footprints is shown below. **(C)** Placement of mitochondrial ribosome footprints with respect to the A and P sites of the ribosome based on reads aligning to start and stop codons. A diagram of the placement of 28-nucleotide mitochondrial ribosome footprints is shown below.
(TIF)

S5 Fig Genome-wide views of the plastid translatome and transcriptome in Segments 1 and 14 displayed using different Y-axis scales. The data are expressed as reads per kilobase per million reads mapping to nuclear genome coding sequences (RPKM). Values are the mean ± SEM from three replicates. Intron-containing genes are marked with a superscript *i*. Genes for which RNA levels and translational efficiency were not determined (n.d.) are marked (#). These include intron-containing genes for which the fraction of reads derived from spliced transcripts is uncertain, and *petN*, whose short mRNA is not represented quantitatively in the RNA-seq data. Genes encoding assembly factors are marked with asterisks. **(A)** Translational output, RNA abundance and translational efficiency displayed according to native gene order. Co-transcribed genes are marked with arrows that indicate the direction of transcription. **(B)** Translational output and RNA abundance displayed according to gene function. Genes encoding assembly factors are demarcated from the structural genes with dashed lines.
(TIF)

S6 Fig Data for translational output, mRNA abundance and translational efficiency parsed according to gene function. The data are expressed as reads per kilobase per million reads mapping to nuclear coding sequences (RPKM). Values represent the mean ± SEM from three replicates. Intron-containing genes are marked with a superscript *i*. Genes for which RNA levels and translational efficiency were not calculated are marked with a hashtag (#). These include intron-containing genes for which the fraction of reads derived from spliced transcripts is uncertain, and *petN* whose very short mRNA is not represented quantitatively in the RNA-seq data due to the library protocol. n.d., not determined. **(A)** Genes related to cytochrome *b*_*6*_*f* function. The *pet* genes encode cytochrome *b*_*6*_*f* subunits and the *ccsA* gene encodes a protein involved in heme attachment [98]. **(B)** Genes encoding subunits of the NDH complex. **(C)** Genes encoding ribosomal proteins.
(TIF)

S7 Fig Heat map representation of the results of hierarchical clustering of plastid genes according to their developmental dynamics. Clusters were generated independently from the data for translational output, mRNA level, and translational efficiency. The genes in each cluster are shown in Fig 5A.
(TIF)

S8 Fig Developmental dynamics of processed mRNA isoforms from two polycistronic transcription units whose genes exhibit distinct developmental shifts in translational efficiency. The transcription units and probes used for RNA gel blot hybridizations are shown at top. Lanes contain an equal mass of total RNA, as illustrated by the methylene blue-stained blots shown below. RNAs were extracted from aliquots of the leaf lysates used for the ribosome footprint preparation prior to RNAse I addition, and therefore suffered slight degradation. The *rps14* gene is represented by several small transcripts that are over-represented with respect to the precursor at early developmental stages. The *psaI* gene is represented on a monocistronic mRNA that accumulates preferentially in segments 4 and 9 and on the polycistronic primary transcript.
(TIF)

S1 Table List of DNA oligonucleotides used in the rRNA depletion step of Ribo-seq library preparation. (XLSX)

S2 Table Mapping statistics of leaf segment data. (XLSX)

S3 Table RNA-seq data at splice junctions of plastid genes. (XLSX)

S4 Table Values for translational output, mRNA level and translational efficiency from the leaf segment data. (XLSX)

S5 Table Hierarchical clustering of the leaf segment data. (XLSX)

S6 Table Values for translational output, mRNA level and translational efficiency from the mesophyll and bundle sheath data. (XLSX)

S7 Table Ribo-seq and RNA-seq data at sites of plastid RNA editing. (XLSX)
