# Supplementary figures and images for: Dynamics of Chloroplast Translation during Chloroplast Differentiation in Maize
Source: PLoS Genet. 2016 Jul 14;12(7):e1006106. doi: 10.1371/journal.pgen.1006106 (PMC4945096; doi:10.1371/journal.pgen.1006106)

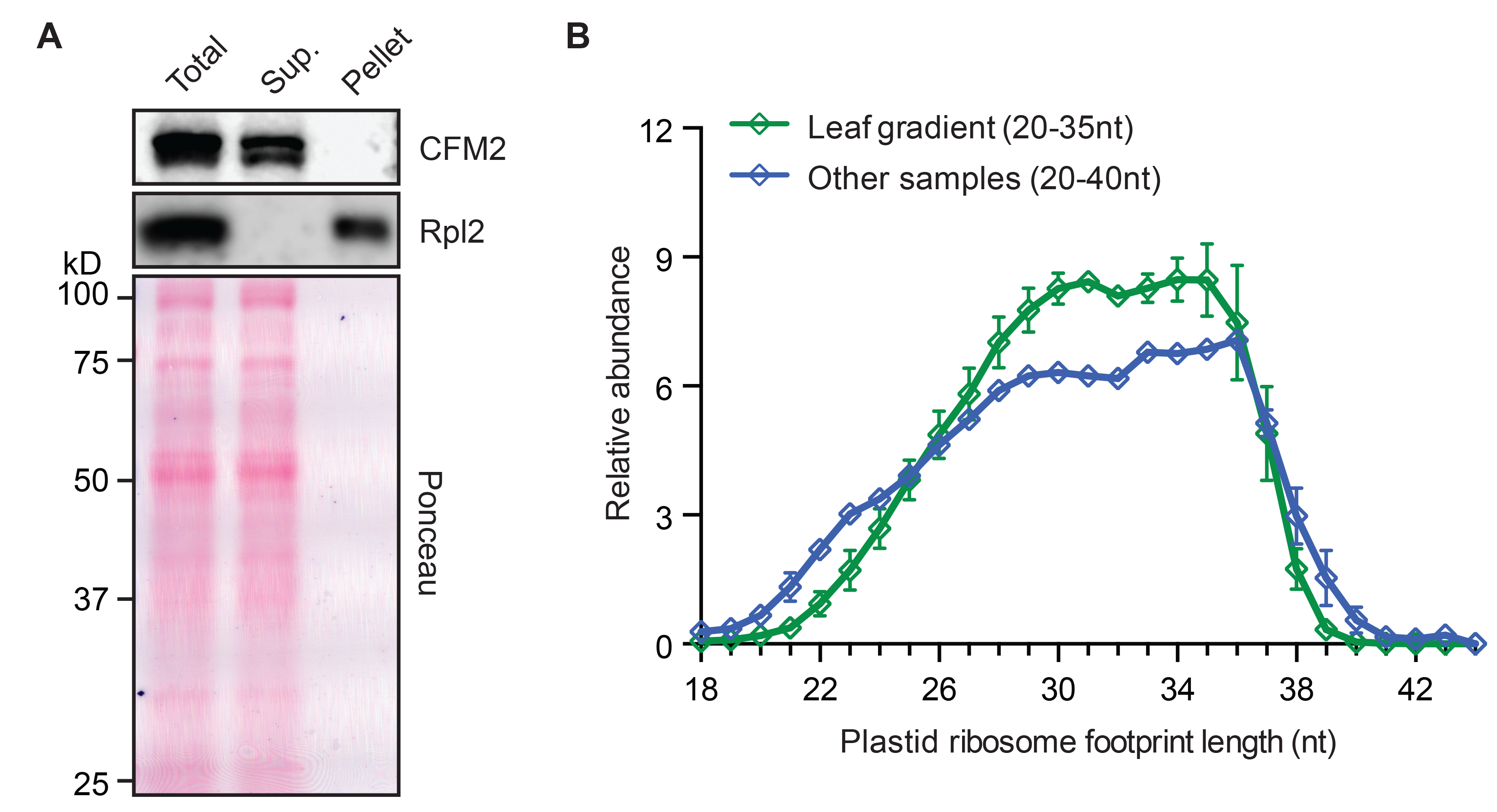

Supplement: S1 Fig — (A) Immunoblots showing that a marker for chloroplast ribosomes (Rpl2) was highly enriched in the pellet after sedimentation of nuclease-treated extract through a sucrose cushion, whereas a subunit of a ~600 kDa group II intron RNP (CFM2) [16] remained in the supernatant. An equal proportion of the starting material, the supernatant above the sucrose cushion, and the pellet fraction was analyzed. (B) Comparison of size distribution of chloroplast ribosome footprints resulting from two different size selection strategies. The experiments in this study used gel purified RNA fragments between approximately 20 and 35-nt (green). A pilot experiment used gel-purified RNA fragments between approximately 20 and 40-nt (blue). The size distribution of the sequence reads was nonetheless similar. (TIF) [file pgen.1006106.s001.tif]

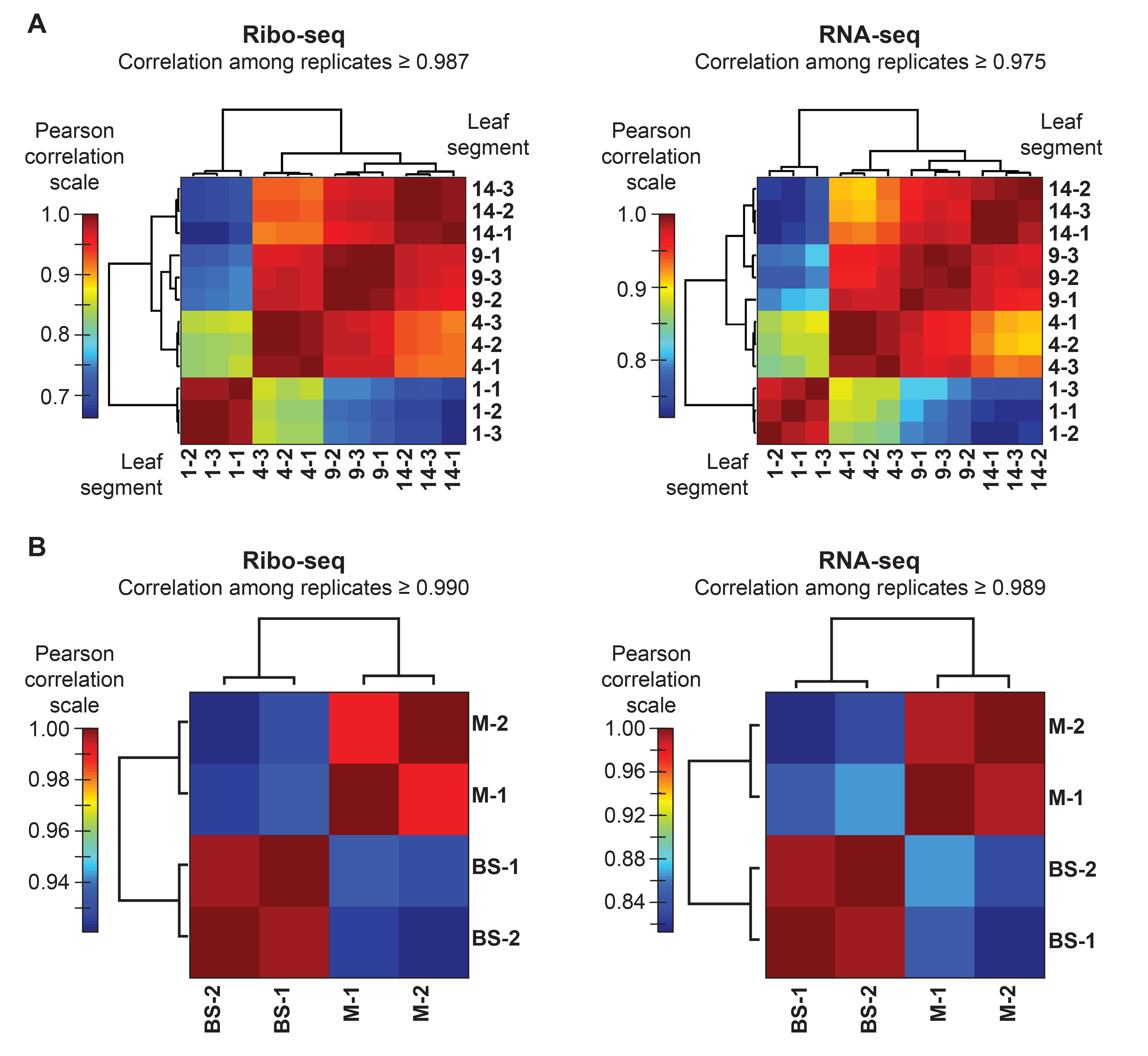

Supplement: S2 Fig — Pearson correlation coefficients for each sample pair combination were calculated using log10 of RPKM values for each protein-coding gene in the chloroplast genome. The correlation coefficients were used as the input for hierarchical clustering. The replicate number of each leaf segment sample is indicated after the hyphen. (A) Leaf segment data. (B) Bundle sheath (BS) and mesophyll (M) data. (TIF) [file pgen.1006106.s002.tif]

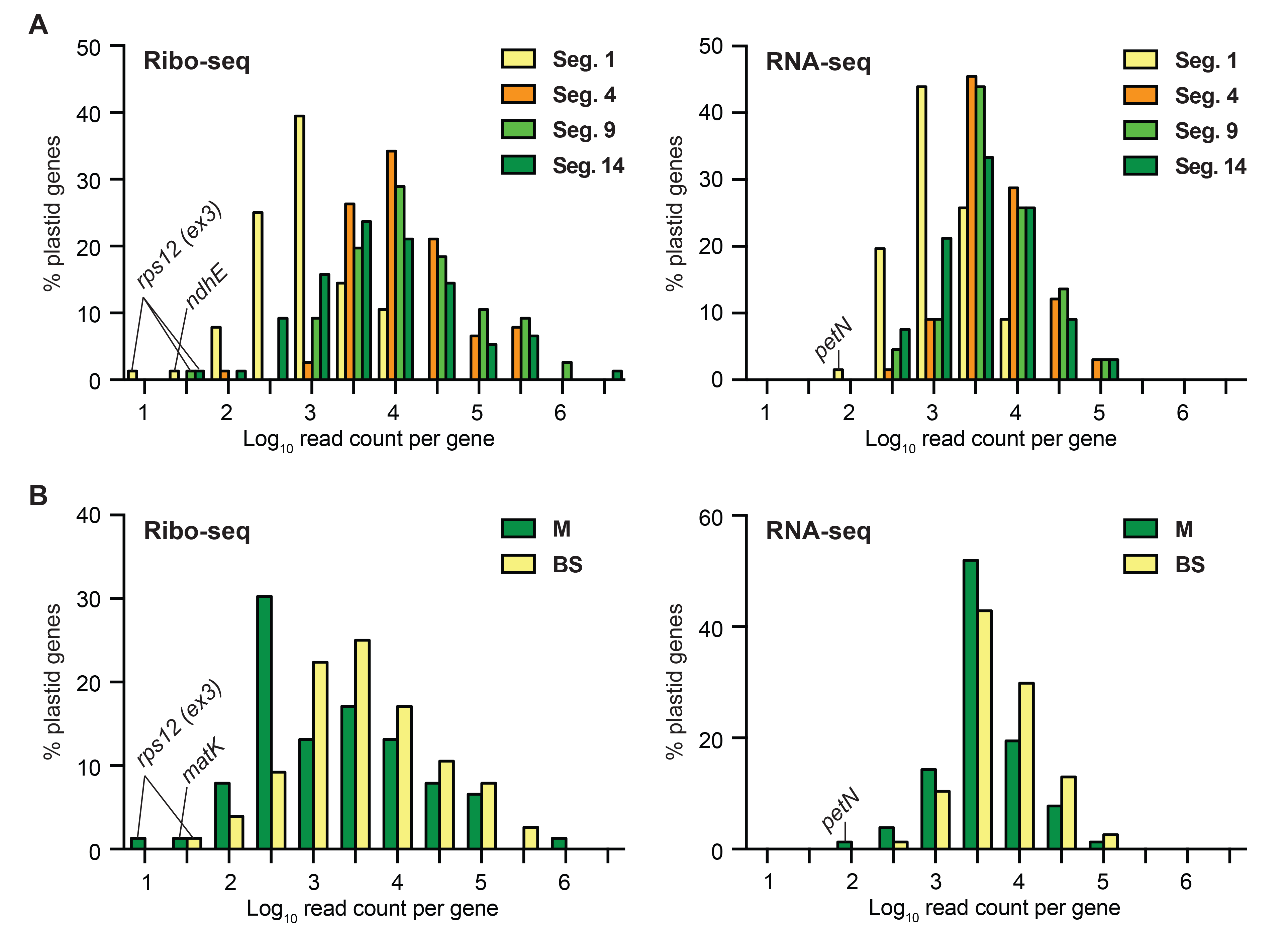

Supplement: S3 Fig — The values displayed are the mean from replicate assays. The identities of genes with low read counts are indicated. The petN mRNA is under represented in the RNA-seq data due to its small size, which is below the cut-off used for library preparation. (A) Read counts/gene for leaf gradient samples. (B) Read counts/gene for bundle sheath (BS) and mesophyll (M) samples. (TIF) [file pgen.1006106.s003.tif]

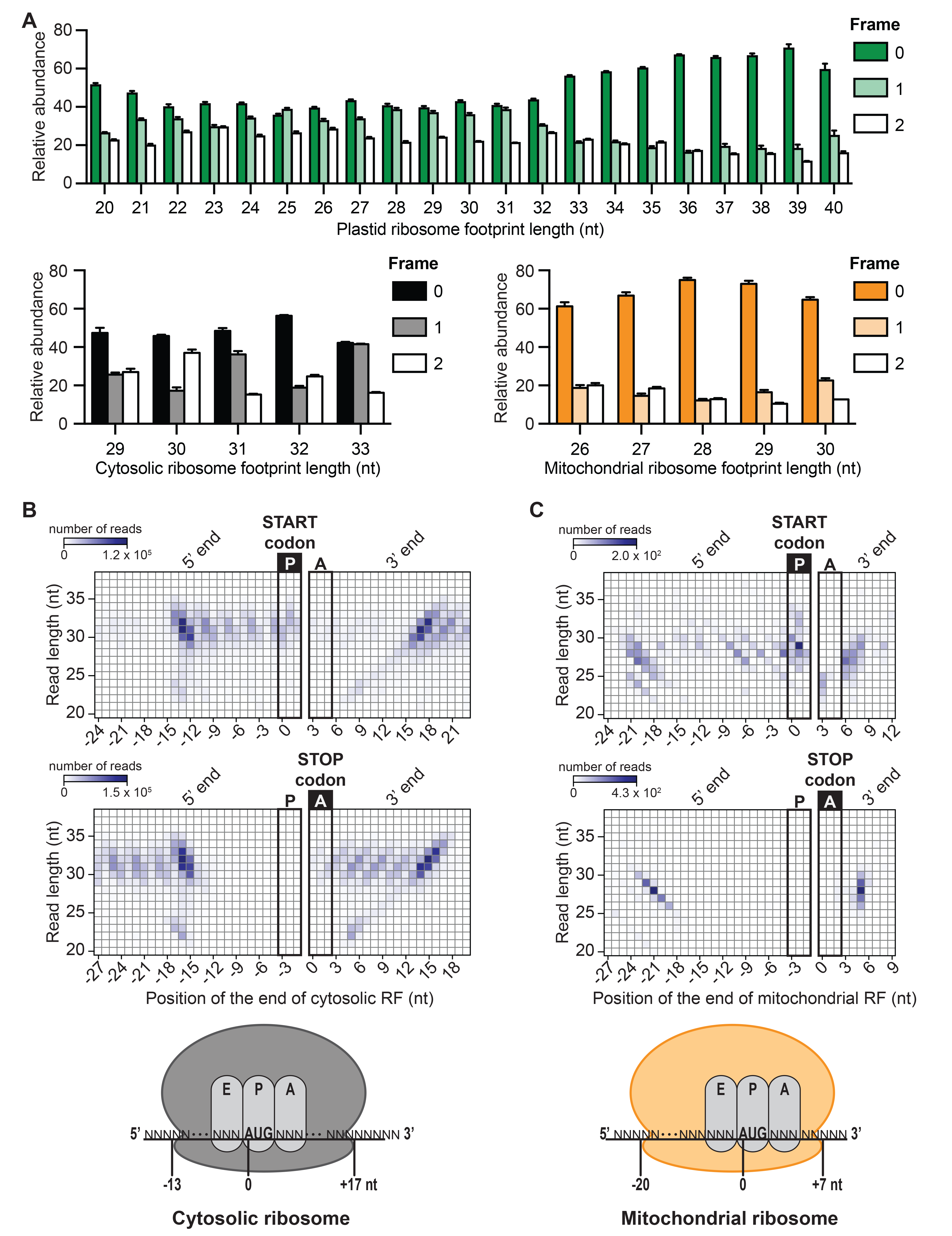

Supplement: S4 Fig — The data plotted come from the twelve leaf gradient samples. (A) Three-nucleotide periodicity of P site position as a function of ribosome footprint length in the plastid, cytosol, and mitochondrion. Values shown are the mean ± SEM. The position of the P site in each footprint was inferred from the footprint size distributions at start/stop codons (Fig 2E and S4B-C). (B) Placement of cytosolic ribosome footprints with respect to the A and P sites of the ribosome based on reads aligning to start and stop codons. A diagram of the placement of 31-nucleotide cytosolic ribosome footprints is shown below. (C) Placement of mitochondrial ribosome footprints with respect to the A and P sites of the ribosome based on reads aligning to start and stop codons. A diagram of the placement of 28-nucleotide mitochondrial ribosome footprints is shown below. (TIF) [file pgen.1006106.s004.tif]

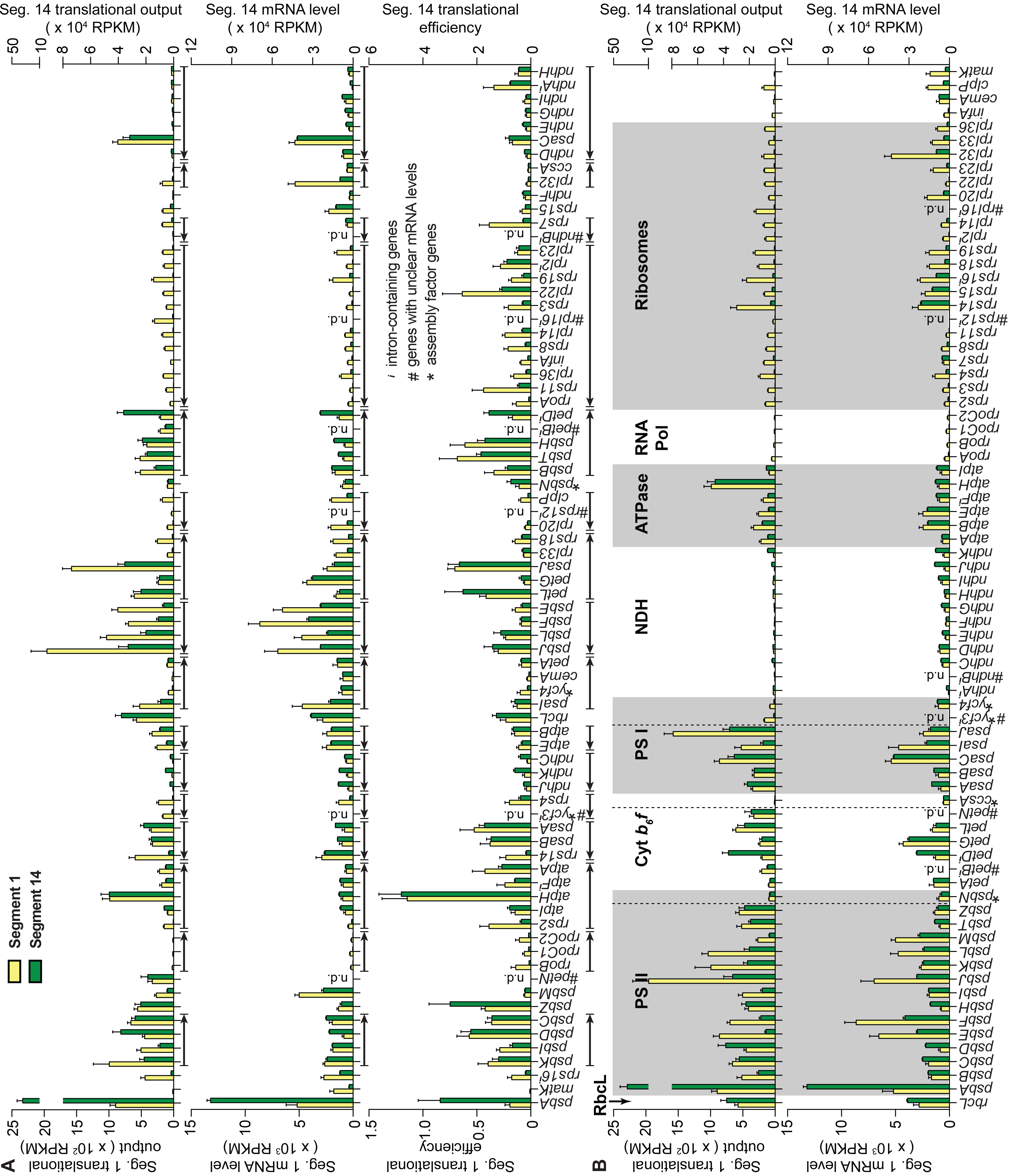

Supplement: S5 Fig — The data are expressed as reads per kilobase per million reads mapping to nuclear genome coding sequences (RPKM). Values are the mean ± SEM from three replicates. Intron-containing genes are marked with a superscript i. Genes for which RNA levels and translational efficiency were not determined (n.d.) are marked (#). These include intron-containing genes for which the fraction of reads derived from spliced transcripts is uncertain, and petN, whose short mRNA is not represented quantitatively in the RNA-seq data. Genes encoding assembly factors are marked with asterisks. (A) Translational output, RNA abundance and translational efficiency displayed according to native gene order. Co-transcribed genes are marked with arrows that indicate the direction of transcription. (B) Translational output and RNA abundance displayed according to gene function. Genes encoding assembly factors are demarcated from the structural genes with dashed lines. (TIF) [file pgen.1006106.s005.tif]

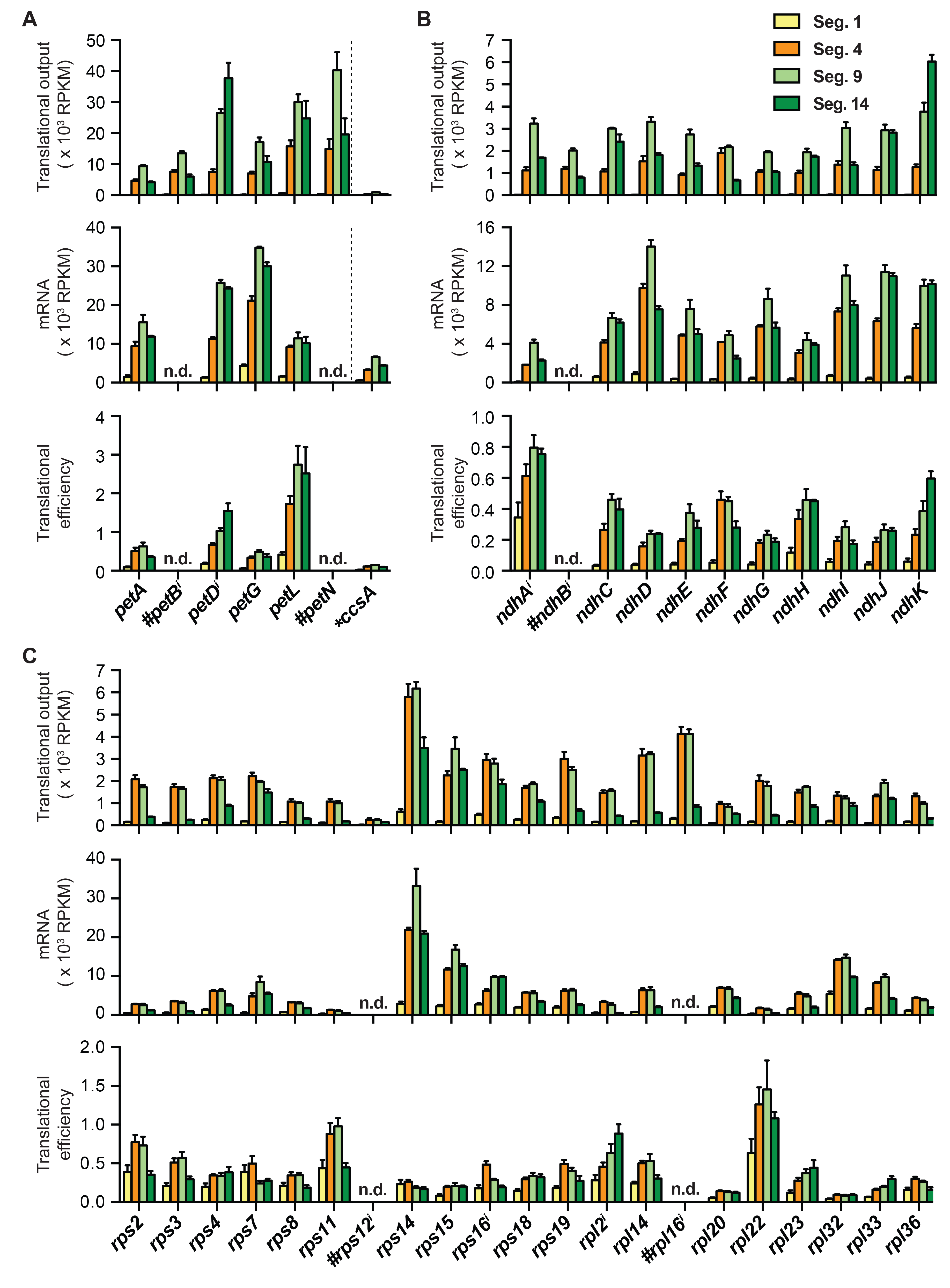

Supplement: S6 Fig — The data are expressed as reads per kilobase per million reads mapping to nuclear coding sequences (RPKM). Values represent the mean ± SEM from three replicates. Intron-containing genes are marked with a superscript i. Genes for which RNA levels and translational efficiency were not calculated are marked with a hashtag (#). These include intron-containing genes for which the fraction of reads derived from spliced transcripts is uncertain, and petN whose very short mRNA is not represented quantitatively in the RNA-seq data due to the library protocol. n.d., not determined. (A) Genes related to cytochrome b6f function. The pet genes encode cytochrome b6f subunits and the ccsA gene encodes a protein involved in heme attachment [98]. (B) Genes encoding subunits of the NDH complex. (C) Genes encoding ribosomal proteins. (TIF) [file pgen.1006106.s006.tif]

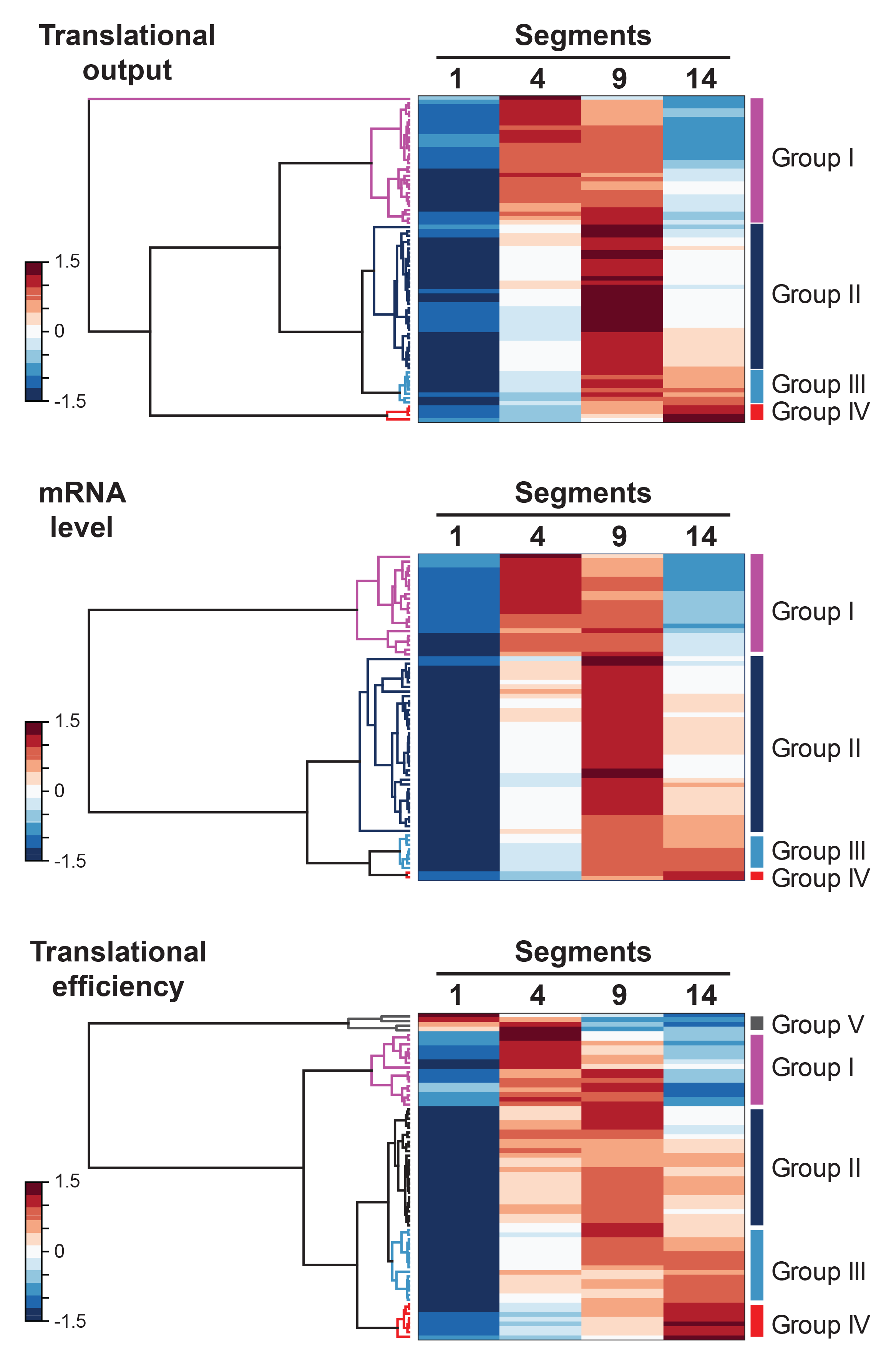

Supplement: S7 Fig — Clusters were generated independently from the data for translational output, mRNA level, and translational efficiency. The genes in each cluster are shown in Fig 5A. (TIF) [file pgen.1006106.s007.tif]

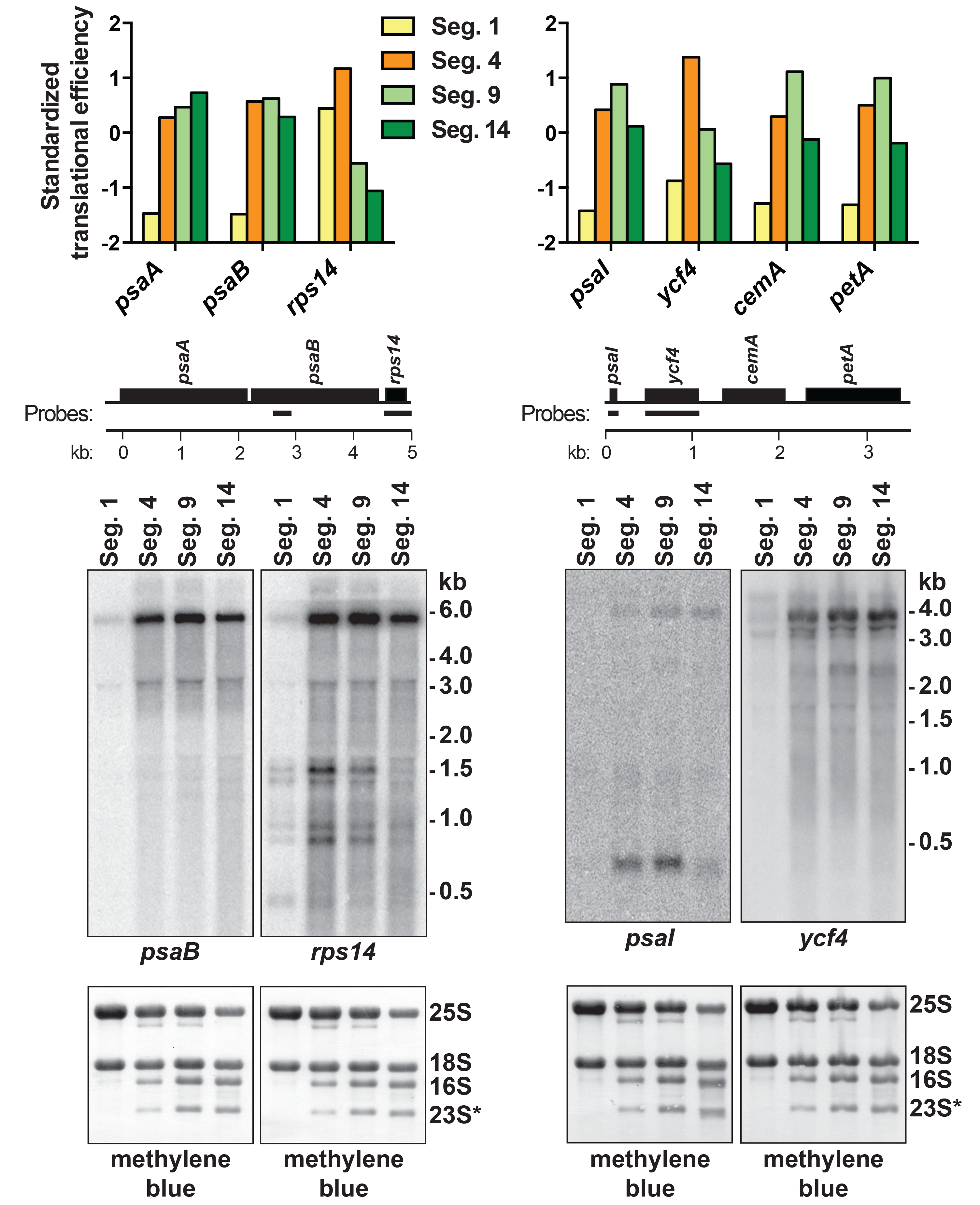

Supplement: S8 Fig — The transcription units and probes used for RNA gel blot hybridizations are shown at top. Lanes contain an equal mass of total RNA, as illustrated by the methylene blue-stained blots shown below. RNAs were extracted from aliquots of the leaf lysates used for the ribosome footprint preparation prior to RNAse I addition, and therefore suffered slight degradation. The rps14 gene is represented by several small transcripts that are over-represented with respect to the precursor at early developmental stages. The psaI gene is represented on a monocistronic mRNA that accumulates preferentially in segments 4 and 9 and on the polycistronic primary transcript. (TIF) [file pgen.1006106.s008.tif]
